# Effects of phthalate exposure on asthma may be mediated through alterations in DNA methylation

**DOI:** 10.1186/s13148-015-0060-x

**Published:** 2015-03-15

**Authors:** I-Jen Wang, Wilfried JJ Karmaus, Su-Lien Chen, John W Holloway, Susan Ewart

**Affiliations:** Department of Pediatrics, Taipei Hospital, Ministry of Health and Welfare, #127, Su-Yuan Road, Hsin-Chuang Dist 242 New Taipei City, Taiwan; Institute of Environmental and Occupational Health Sciences, College of Medicine, National Yang-Ming University, Taipei, 112 Taiwan; Department of Health Risk Management, China Medical University, Taichung, 404 Taiwan; Division of Epidemiology, Biostatistics, and Environmental Health, School of Public Health, University of Memphis, Memphis, 38152 USA; Genomics, BioSci & Tech, Taipei, 221 Taiwan; Clinical and Experimental Sciences, Faculty of Medicine, and NIHR Respiratory Biomedical Research Unit, University of Southampton, Southampton, S016 6YD UK; Human Development and Health, Faculty of Medicine, University of Southampton, Southampton, SO16 6YD UK; Department of Large Animal Clinical Sciences, Michigan State University, East Lansing, 48824 MI USA

**Keywords:** DEHP exposure, DNA methylation, *TNFα*, Asthma, Quantitative PCR

## Abstract

**Background:**

Phthalates may increase the asthma risk in children. Mechanisms underlying this association remain to be addressed. This study assesses the effect of phthalate exposures on epigenetic changes and the role of epigenetic changes for asthma. In the first step, urine and blood samples from 256 children of the Childhood Environment and Allergic diseases Study (CEAS) were analyzed. Urine 5OH-MEHP levels were quantified as an indicator of exposure, and asthma information was collected. DNA methylation (DNA-M) was measured by quantitative PCR. In the screening part of step 1, DNA-M of 21 potential human candidate genes suggested by a toxicogenomic data were investigated in 22 blood samples. Then, in the testing part of step 1, positively screened genes were tested in a larger sample of 256 children and then validated by protein measurements. In step 2, we replicated the association between phthalate exposure and gene-specific DNA-M in 54 children in the phthalate contaminated food event. In step 3, the risk of DNA-M for asthma was tested in 256 children from CEAS and corroborated in 270 children from the Isle of Wight (IOW) birth cohort.

**Results:**

Differential methylation in three genes (*AR*, *TNFα*, and *IL-4*) was identified through screening. Testing in 256 children showed that methylation of the *TNFα* gene promoter was lower when children had higher urine 5OH-MEHP values (*β* = −0.138, *P* = 0.040). Functional validation revealed that *TNFα* methylation was inversely correlated with TNFα protein levels (*β* = −0.18, *P* = 0.041). In an additional sample of 54 children, we corroborated that methylation of the *TNFα* gene promoter was lower when urine 5OH-MEHP concentrations were higher. Finally, we found that a lower methylation of 5′CGI region of *TNFα* was associated with asthma in 256 CEAS children (OR = 2.15, 95% CI = 1.01 to 4.62). We replicated this in 270 children from the IOW birth cohort study. Methylation of the CpG site cg10717214 was negatively associated with asthma, when children had ‘AA’ or ‘AG’ genotype of the *TNFα* single nucleotide rs1800610.

**Conclusions:**

Effects of phthalate exposure on asthma may be mediated through alterations in DNA methylation.

**Electronic supplementary material:**

The online version of this article (doi:10.1186/s13148-015-0060-x) contains supplementary material, which is available to authorized users.

## Background

In 2011, the Food and Drug Administration (FDA) in Taiwan discovered that manufacturers had replaced expensive natural emulsifiers with di-(2-ethylhexyl) phthalate (DEHP) in numerous food and drinks for several decades [1]. This ‘contaminated food event’ caused uncertainties among the general public and resulted in scientific assessments. Phthalate has been described as an endocrine disruptor. Epidemiological studies suggested an association between exposure to DEHP and increased prevalence of asthma or wheezing [2-4]. Pulmonary physiological data suggests that DEHP may promote and aggravate allergic asthma by an adjuvant effect [5]. DEHP is also considered to promote a deviation of Th2 response (related to allergy) by suppressing *IFN-α/IFN-β* gene expression and by modulating interleukin 4 (IL-4) and immunoglobulin E (IgE) production [6,7]. In an earlier cohort study, we demonstrated that exposure to phthalate was positively correlated with serum IgE levels with higher risk of atopic disorders in children [8]. In addition, we reported that urine DEHP metabolite levels were higher in Taiwanese children compared to US and German children [8-10].

**Figure 1:**
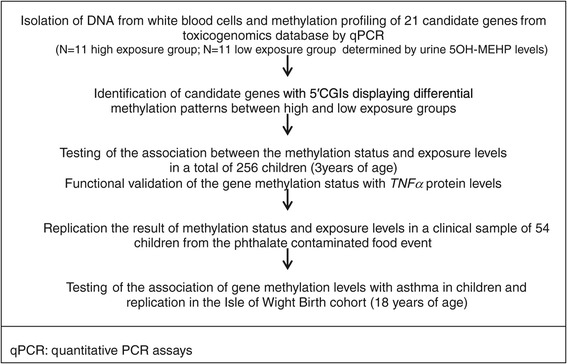
**Study flow chart.**

Animal studies have identified that phthalates, among other endocrine disruptors, may modify epigenetic marks [11]. There are numerous reports of epigenetic modifications after environmental toxicant exposures, but most have not been directly linked to a disease endpoint [11,12]. Although data generated from clinical specimens after environmental exposure monitoring are limited, epigenetic changes are considered to mediate associations of environmental exposures with asthma [13,14]. This motivated us to investigate potential epigenetic effects of phthalate exposures on DNA methylation (DNA-M) and then test the effect of the methylation on asthma. In the first step, urine and blood samples from 256 children of the Childhood Environment and Allergic diseases Study (CEAS) were analyzed. In the screening part of step 1, DNA-M of 21 potential human candidate genes suggested by a toxicogenomic database due to suspected associations between phthalates and DNA-M were screened in 22 blood samples. In the testing part of step 1, three positively screened genes were tested in all 256 children and then validated by protein measurements. In step 2, we replicated the association between phthalate exposure and gene-specific DNA-M in 54 children whose parents took them a clinical examination because of DEHP contaminated food events in Taiwan in 2011. Urine and blood specimens were analyzed in these children. In step 3, the risk of DNA methylation for asthma was tested in 256 children from CEAS and corroborated in 270 children from the Isle of Wight (IOW) birth cohort [15] (Figure 1). Results from cross-sectional investigation between methylation of multiple genes and asthma in the IOW birth cohort have been published elsewhere. We hypothesize that the effect of phthalate exposures on asthma is mediated through alterations in DNA methylation.

## Results

### Three genes with differential methylation were selected for testing in a larger sample

After screening of 21 candidate genes by quantitative polymerase chain reaction (qPCR) (Table 1, Additional file 1: Table S1), differential methylation statuses for high and low exposure groups were identified for three genes (*androgen receptor* (*AR*), *tumor necrosis factor α* (*TNF*α), and *IL*-*4*). There were no significant differences in 18 other genes (*ESR1, ESR2, PGR, ESRRG, PPARA, PPARG, THRB, CYP1A1, CYP1B1, CYP19A1, VEGFA, MAPK1, MAPK3, STAT3, LIF, NR1I2, LAMP,* and *TFF1*) (Additional file 1: Table S2). The three genes were selected for further testing in a larger sample of 256 children. The methylation percentages of three candidate genes in 22 samples are presented in Table 1. Basic demographic information of the study population is provided in Table 2. With the exception of maternal education, there were no significant differences between those with complete blood samples (*N* = 256) and those without (*N* = 453).

**Table 1:** **Three candidate genes with differential methylation between low and high DEHP exposure in the screening**

**Gene gene ID**	**CpG island location**	**Assay details** ^**b**^
	**TSS position**	**Met%** ^**a**^ **(mean ± SD) of low and high DEHP exposure**
*AR* (androgen receptor) (367)	ChrX: 66763684-66764077	Catalog number: 335002 EPHS115070-1A
		SABiosciences CpG Island ID: 115070
		CpG island location: ChrX: 66763684-66764077
		Assay position (central point): ChrX: 66763921
		PCR product size: 171
		NCBI build number: 37
	66763873	Low *vs.* high exposure:
		35.73 ± 31.76 *vs.* 14.95 ± 17.23
		*P* = 0.071 by qPCR
*TNFα* (tumor necrosis factor α) 7124)	Chr6: 31543344-31544344	Catalog number: 335002MePH80050-1A
		SABiosciences CpG Island ID: custom design
		CpG island location: Chr6: 31543344-31544344
		Assay position (central point): Chr6: 31543573
		PCR product size: 294
		*(The sequence is in the Supplement)*
		NCBI build number: 37
	31543350	Low *vs.* high exposure:
		42.65 ± 26.36 *vs.* 35.61 ± 27.13
		*P* = 0.036 by qPCR
*IL-4* (Interleukin-4) (16189)	Chr5: 132035956-132036176	Pyrosequence primer info:
		F:GTTGATTGGTTTTAAGTGATTGATAATT
		R:Biotin-ATACCAAATAAATACTCACCTTCTACT
		S:TTTTTGTTTTTTTTGTTAGTATGT
		Sequence to analysis:
		GTYGGTAATTTTGTTTAYGGATATAAGTGYGATATTATTTTATAGGAGATTATTA
	132040541	Low *vs.* high exposure:
		89.36 ± 3.58 *vs.* 89.36 ± 7.56
		*P* = 0.249 by pyrosequencing

^a^Met%: percentage of methylated cytosine in the CGIs; ^b^ *AR* and *TNFα* results were identified by EpiTect Methyl II qPCR assay. Since there was no commercial kit for *IL-4* DNA methylation by qPCR, *IL-4* results were obtained by pyrosequencing method via QIAGEN PyroMark Q24 instrument.

**Table 2:** **Population characteristics**

**Category**	**Subjects with urine and blood specimens**	**Initial cohort with urine specimens**	***P*** **value**
	**(** ***N*** **= 256)**	**(** ***N*** **= 453)**	
Gender (male) (%)	53.5	57.7	0.31
Prematurity <37 week (%)	92.1	91.0	0.66
Maternal age ≥34 years (%)	17.3	17.8	0.89
Maternal history of atopy (%Yes)	42.6	35.1	0.10
Maternal education ≥ college (%)	40.3	30.8	0.03*
Breast feeding (%Yes)	79.8	76.4	0.39
ETS exposure (%Yes)	40.3	46.2	0.10
Family income per year >1,500,000 (NT dollars) (%)	8.7	8.0	0.33
Asthma (%Yes)	25.4	26.9	0.89

*P<0.05.

### Inverse association of TNFα methylation status with exposure in a larger sample

In a larger sample of 256 children, the levels of the geometric means (standard deviations (SDs)) of 5OH-MEHP, MEP, MBP, and MBzP were 6.80 (3.40), 14.29 (3.87), 5.49 (3.95), and 0.70 (3.04) ng/mL, respectively. After adjusting for confounders, the methylation of the 5′CGI region of the *TNFα* gene was inversely associated with Ln-5OH-MEHP (*β* = −0.138, *P* = 0.040) and Ln-MBzP (*β* = −0.144, *P* = 0.032) (Table 3). Since both Ln-5OH-MEHP and Ln-MBzP were correlated (Spearman’s *r* = 0.139, *P* = 0.027) and 5OH-MEHP presents a high fraction of the metabolites, we decided to focus on 5OH-MEHP. The association of *AR* methylation percentage with Ln-5OH-MEHP levels and *IL*-*4* methylation percentage with Ln-5OH-MEHP levels failed to reach a level of statistical significance after adjusting for urine creatinine, white blood cell proportion, gender, age, maternal education, and environmental tobacco smoke exposure (*β* = −0.103, *P* = 0.237; *β* = −0.217, *P* = 0.147, data not shown).

**Table 3:** **The association of log-transformed urine phthalate levels and standardized regression coefficients βeta for** ***TNFα*** **methylation**

**Urine phthalate metabolites** ^**b**^	**Ln-MEP**	***P*** **value**	**Ln-MBP**	***P*** **value**	**Ln-MBzP**	***P*** **value**	**Ln-5OH-MEHP**	***P*** **value**
*TNFα* (Met%)								
Crude *β*	−0.009	0.887	−0.057	0.367	−0.154	0.014*	−0.175	0.005*
Adjusted *β* ^a^	−0.045	0.508	−0.044	0.534	−0.144	0.032*	−0.138	0.040*

MEP, monoethyl phthalate; MBP, monobutyl phthalate; MBzP, monobenzyl phthalate; 5OH-MEHP, mono(2-ethyl-5-hydroxyhexyl)phthalate; Met%, methylation percentage. ^a^Adjustment for urine creatinine, white blood cell proportion, gender, age, maternal education, and environmental tobacco smoke exposure; ^b^The main urine phthalate metabolite levels measured in this study included MEP, MBP, MBzP, and 5OH-MEHP.*P < 0.05.

To validate whether the *TNFα* 5′CGI methylation status affects its protein production, we analyzed plasma TNFα protein level in 256 children. The mean level was 901.78 pg/mL (SD: 11.51 pg/mL). We found that TNFα protein level was inversely associated with *TNFα* methylation percentage (*β* = −0.18, *P* = 0.041; Additional file 1: Figure S2).

### Phthalate-methylation associations in a clinical sample

To test if the association between phthalate exposure and DNA methylation identified in the high exposure cohort, we evaluated the correlation between *TNF*α methylation and 5OH-MEHP level in 54 children who attended a clinic due to phthalate-contaminated food event. In these children, the geometric mean level of 5OH-MEHP was 9.51 ng/mL (SD: 6.47 ng/mL). *TNF*α methylation percentage showed an inverse association with 5OH-MEHP level (*β* = −0.246, *P* = 0.168), though it failed to reach statistical significance.

### Phthalate exposure was positively associated with asthma

To test the association of phthalates with asthma, the cohort of 256 children were divided into three groups based on tertiles of urine 5OH-MEHP levels. After adjusting for gender, age, prematurity, maternal history of atopy, maternal education, and environmental tobacco smoke exposure, urine 5OH-MEHP levels were significantly associated with asthma (OR = 2.17, 95% CI = 1.03 to 4.56) for the highest tertile (>19.33 ng/mL) compared with the lowest tertile (<4.14 ng/mL; Table 4). There was no significant association between MBzP and asthma.

**Table 4:** **The association of phthalate exposure with asthma in children**

**Urine 5OH-MEHP levels (ng/mL)**	**Subjects**	**Asthma**	**Non-asthma**	**OR**	**Adjusted OR** ^**a**^
	**(** ***N*** **= 256)**	**(** ***N*** **= 56)**	**(** ***N*** **= 200)**	**(95% CIs)**	**(95% CIs)**
>19.33	54	18(32.1)	36(18.0)	2.30(1.15 to 4.63)*	2.17(1.03 to 4.56)*
4.14 to 19.33	45	10(17.9)	35(17.5)	1.12(0.58 to 2.97)	1.29(0.54 to 3.06)
<4.14	157	28(50.0)	129(64.5)	1	1

^a^Adjustment for gender, age, prematurity, maternal history of atopy, maternal education, and environmental tobacco smoke exposure. *P<0.05.

### Lower methylated TNFα 5′CGI was associated with asthma

To analyze the association of *TNF*α 5′CGI methylation with asthma in CEAS cohort, we first compared mean methylation levels. A lower *TNF*α methylation percentage was found in children with asthma (mean ± SD: 31.53 ± 25.96%) in contrast to children without (41.14 ± 26.88%, *P* = 0.018). For logistic regression analysis, we dichotomized the methylation into low and high. After adjusting for gender, age, prematurity, maternal history of atopy, maternal education, and environmental tobacco smoke exposure, *TNFα* 5′CGI of children in the lower methylated group were found to be positively associated with asthma (OR = 2.15, 95% CI = 1.01 to 4.62) compared to the higher methylated group (Table 5).

**Table 5:** **The association of** ***TNFα*** **5′CGI methylation status and asthma in children**

	**Asthma**	**Non-asthma**	**Subjects**	**OR**	**Adjusted OR** ^**b**^
	**(** ***N*** **= 56)**	**(** ***N*** **= 200)**	**(** ***N*** **= 256)**	**(95% CIs)**	**(95% CIs)**
Lower methylated *TNFα* 5′CGI^a^	35(62.5)	93(46.5)	128(50.0)	1.92(1.04 to 3.52)*	2.15(1.01 to 4.62)*
Higher methylated	21(37.5)	107(53.5)	128(50.0)	1	1

^a^ *TNFα* methylation status was dichotomized into lower and higher methylated group with cutoff value of median of promoter methylation percentage; ^b^Adjustment for gender, age, prematurity, maternal history of atopy, maternal education, and environmental tobacco smoke exposure. *P<0.05.

To test whether findings regarding DNA-M and asthma could be replicated, we used DNA methylation data available from the IOW UK birth cohort. We identified three cytosine-phosphate-guanine sites (CpGs) within the Illumina Infinium HumanMethylation450 BeadChips data of the IOW birth cohort that were included in the PCR product sequence of the *TNFα* methylation in the Taiwanese study (cg04425624, cg10717214, and cg10650821). Since the Taiwanese and the UK children have different genetic backgrounds, we additionally tested the *TNFα* single nucleotide polymorphism (SNP) rs1800610, an asthma candidate SNP. The interaction of the SNP rs1800610 and methylation of cg10717214 on risk of asthma showed a statistically significant effect. When the methylation level of cg10717214 was low and the genotype was AA or AG, the relative risk (RR) of asthma was higher (8% methylation: RR = 4.28, *P* = 0.008; 10% methylation: RR = 2.25, *P* = 0.015, Additional file 1: Figure S3).

### Does TNFα 5′CGI methylation link phthalate exposure with asthma?

Our study design initiated from phthalate exposure and tested potential changes in DNA methylation. The association of phthalate exposure and *TNFα* 5′CGI methylation with asthma was assessed in the last step. This flow of assessments protected us from reverse causation, namely that phthalate exposure and the methylation of the 21 candidate genes resulted from different immune and metabolic responses related to asthma.

In addition, to estimate whether *TNFα* 5′CGI methylation is an intervening variable between 5OH-MEHP and asthma, we conducted a mediation analysis. The mediation analyses incorporated the different regression coefficients (5OH-MEHP and *TNFα* 5′CGI, Additional file 1: Figure S4). It is estimated that 20% of the total effect of 5OH-MEHP on asthma is mediated by *TNFα* 5′CGI.

## Discussion

This is the first epidemiological study linking phthalate exposure with DNA methylation and asthma in humans. We demonstrated that higher exposure to phthalates is related to lower DNA methylation of *TNFα*, which in turn is associated with higher risk of asthma in children. In addition, we showed that the protein levels of *TNFα* were inversely related to phthalate exposures. The association between phthalate exposures and *TNFα* methylation was replicated in another 54 children who attended a clinic due to a phthalate contaminated food event. The role of DNA methylation of *TNFα* with asthma was further replicated in the Isle of Wight birth cohort for children with a specific *TNFα* polymorphism. In addition, a mediation analysis demonstrated that *TNFα* 5′CGI may be a potential epigenetic link between phthalate and asthma.

We chose 5OH-MEHP as an indicator of DEHP exposure because it represents high fraction of all metabolites. In addition, we replicated our findings in another sample of children in the DEHP contaminated food event. *TNFα* methylation percentage in this group also showed an inverse correlation with 5OH-MEHP level. These findings support our hypothesis that phthalate exposure may affect *TNFα* methylation. DEHP has been demonstrated to induce TNFα release and macrophage differentiation in murine monocyte-macrophage cell line [16]. The study by Lee *et al*. indicates that DEHP-induced allergy-related cytokine (IL-4 and TNFα) production by human mast cell line-1 cells [17]. Win-Shwe *et al*. reported that exposure to DEHP may up-regulate *TNFα* mRNA expression levels in mice [18]. Further, dendritic cells treated with endocrine disrupters were reported to increase *TNFα* expression and epigenetic change at the *TNFα* gene locus [19]. In the screening, we also found that *AR* methylation was weakly related to phthalate exposure (*P* = 0.071). However, in the large sample, *AR* failed to display significant differences in methylation after adjusting for potential confounders. Since different cell types of white blood cells might be responsible for the DNA methylation changes, we have adjusted white blood cell proportion in the model to exclude the confounding effect. However, we did not conduct differential blood cell counts or flow cytometry. Hence, we have no data on whether specific white blood cells were highly methylated and established the link with asthma.

A lower methylation of *TNFα* 5′CGI was positively associated with asthma. Moreover, *TNFα* methylation and TNFα protein levels tested in 256 children showed an inverse relationship. Hence, high phthalate exposure might lead to lower methylation of *TNFα* 5′CGI, which in turn is associated with increased protein production. We speculate that an increased TNF*α* protein levels may trigger the development of allergic inflammation. *TNFα* has been implicated in airway inflammation and hyperresponsiveness and the regulation of immune cells, hallmark features of asthma [20]. It may also up-regulate the high affinity IgE receptor and IgE-dependent activation of airway smooth muscle cells [21]. Genetic variants of *TNFα* have been associated with development of asthma, total serum IgE level in asthma patients, and severity of asthma [22,23]. We believe that better understanding of the mechanism of *TNFα* methylation after environmental exposures may shed new light on improved therapeutic strategies for asthma.

There are some limitations of this study. First, our study was limited by the use of a toxicogenomic-based candidate gene approach. The candidate gene approach was based on the prior knowledge about functional aspects of possible candidates, which may lead to an information bottleneck. However, instead of only one candidate gene, we selected 21 candidate genes from a published toxicogenomic database for screening and validation. Although this approach adds to biological plausibility to our study and is more cost-effective than the epigenome-wide approach, we did not conduct a systematic screening of all genes that are potentially affected by phthalate exposure. However, our results demonstrate agreement between *TNFα* methylation and its protein expression. This further supports the role of this newly discovered epigenetic marker. Second, we did not conduct a systematic approach of all CpG sites in the 21 genes but focused on qPCR method of CpG islands in promoter regions. Hence, we cannot exclude that other CpG sites and CpG of additional genes are involved linking phthalate exposure and asthma. Second, we did not investigate whether the methylation of the 21 genetic candidates could have affected by methylation quantitative loci. Third, we did not correct the analysis by multiple comparisons because the sample size was not large enough. However, we repeated the findings in two independent samples. For the phthalate body burden, we used another group of children from Taiwan. For the association of DNA methylation with asthma, we used a cohort of British children. The ability to repeat the analyses in two independent samples establishes a higher burden than adjusting for multiple testing. Fourth, our measurements are based on a single early morning urine sample only. However, spot urine samples and 24-h urine samples produced comparable estimates of daily DEHP intake [24]. Even though phthalates have relatively short half-lives, continuous daily exposure results in an exposure scenario that is similar to persistent and bioaccumulative compounds [25]. In addition, if measurement errors occurred, they tend to be towards the null and the effect of exposure would likely to be underestimated. Fifth, we used a cross-sectional design and cannot exclude reverse causation, namely that asthma influences DNA methylation. However, the analytical approach starting with phthalate exposure, followed by an assessment of the exposure-selected effect of DNA methylation on asthma, minimizes the bias of reverse causation. In addition, we estimated the mediating effect of DNA methylation.

One of the strengths of this study is that we used biological specimens to link clinical and environmental exposure data. The use of urine biomarkers analyzed by ultra-performance liquid chromatography coupled with tandem mass spectrometry (UPLC-MS/MS) offered a more direct measure of individual exposure than house dust by integrating ingestion, inhalation, and dermal absorption. Furthermore, asthma was determined by pediatricians instead of by questionnaire alone. Therefore, the misclassification of asthma is low and not related to exposure. Another strength is that we applied a three-step approach: a qPCR method to identify candidate gene methylation and followed by confirming the candidate gene methylation status in a large sample. Finally, we replicated our findings in two independent pediatric populations. This step-wise approach allowed us to detect the most promising candidates cost-effectively.

## Conclusions

In conclusion, we found that phthalate exposures are associated with DNA methylation and that methylation of *TNFα* in turn is associated with asthma. *TNFα* 5′CGI may be a potential epigenetic biomarker for phthalate-induced asthma. Our results contribute to the understanding of the etiology of phthalate-related asthma and may initiate new strategies for early prevention or therapeutic intervention in children exposed to phthalates.

## Methods

### Study population

In 2010, in Taipei, a total of 256 children from CEAS cohort were recruited and urine and blood specimens were collected. Full enrollment into the study required the completion of phthalate exposure monitoring by checking urine phthalate metabolite levels and blood sampling. Parents were interviewed using a standardized questionnaire in the pediatric clinics on birth history, parental age education levels, parental atopy, child environmental exposures, diet habits, and allergic diseases. Written informed consent was obtained from parents. The study protocol was approved by the Institutional Review Board from Taipei Hospital, Ministry of Health and Welfare, and complied with the principles outlined in the Helsinki Declaration.

The replication study included 94 male and 196 female participants, age 18 years of the IOW birth cohort study, who had DNA-M measured at age 18 years, and information on the *TNFα* SNP rs1800610, asthma at age 18, birth order, and duration of breastfeeding [26].

### Determination of cases

In the CEAS cohort, pediatric allergists performed a standardized history and clinical examination on participants at age 3. Asthma was determined by pediatric allergists based on the following three criteria: (i) recurrence of at least two of the three symptoms: cough, wheeze, and shortness of breath within the previous 12 months, (ii) doctor’s diagnosis of asthma with ongoing treatment, and (iii) response to treatment with β2-agonists or inhaled corticosteroids [27]. In the IOW birth cohort, asthma was defined as having ‘ever had asthma’ and either ‘wheezing or whistling in the chest during the previous 12 months’ or ‘current treatment for asthma’.

### Laboratory methods

#### Exposure monitoring

Urine phthalate metabolite levels (5OH-MEHP, MEP, MBP, and MBzP) were measured by UPLC-MS/MS, as described elsewhere [28]. 5OH-MEHP in urine, the major metabolite of DEHP, was used to approximate exposure. 5OH-MEHP levels below the limit of quantification were imputed as 1.36 ng/mL.

#### gDNA isolation and purification

The gDNA was isolated and purified from the whole blood with the QIAGEN Puregene Blood Kit (QIAGEN #158389, QIAGEN, Inc., Valencia, CA, USA). The DNA quality was measured with spectrophotometer and gel electrophoresis.

#### Detection of DNA methylation by pyrosequencing

The bisulfate conversion of DNA was treated with EpiTect® Plus Bisulfite Conversion K it (QIAGEN #59124, QIAGEN, Inc., Valencia, CA, USA). We also used the reference DNA set (included unmethylated DNA and methylated DNA) (QIAGEN #59695, QIAGEN, Inc., Valencia, CA, USA) to monitor the bisulfate conversion efficiency.

The assays were designed by QIAGEN PyroMark Assay Design v2.0 software (QIAGEN, Inc., Valencia, CA, USA). The primer sequence of these assays is listed in the Table 1 and Additional file 1: Table S2.

The PCR reaction composition using PyroMark PCR Kit (QIAGEN #978703, QIAGEN, Inc., Valencia, CA, USA) are HotStarTaq DNA Polymerase, 1× PyroMark PCR Buffer, dNTPs, 1× CoralLoad, 3 mM MgCl_2_, 0.2 uM Primers, 1× Q-solution, and 20 ng bisulfate-converted DNA. The PCR program was performed by the Veriti Thermo Cycler (Life Technology # 4375786, Life Technologies, Carlsbad, CA, USA). The PCR products were checked with gel electrophoresis.

The PCR products were separated into single strands using streptavidin-coated beads. Add PyroMark Gold Q24 Reagents (QIAGEN #970802, QIAGEN, Inc., Valencia, CA, USA) into QIAGEN Cartridge. The sequence signals were generated by PyroMark Q24 instrument and analyzed the sequence peak signal intensity by PyroMark Q24 software v2.0.6.

#### Detection of DNA methylation by real-time PCR

Restriction digestion was performed using the EpiTect Methyl II DNA Restriction Kit (SAB# 335452). Each sample was divided into four tubes and treated with methylation-sensitive (Ms) restriction enzyme, methylation-dependant(Md) restriction enzyme, Ms + Md, and a mock (no enzymes added), respectively (Additional file 1: Figure S5). Reference DNA sets (included unmethylated DNA and methylated DNA) (QIAGEN #59695, QIAGEN, Inc., Valencia, CA, USA) were used to monitor the enzyme digestion efficiency. Following digestion, the remaining DNA in each individual enzyme reaction was quantified by ViiA7 real-time PCR instrument. The assay primers were designed and synthesized by QIAGEN SABioscience (QIAGEN, Inc., Valencia, CA, USA). The assay primers information is listed in Table 1 and Additional file 1: Table S2. The PCR cycling protocol is 95 C, 10 min, repeat 3 cycles of (99 C, 30 s → 72°C, 1 min), and then repeat 40 cycles of (97 C, 15 s, 72 C, 1 min, and detect fluorescence). The relative fractions of methylated and unmethylated DNA are subsequently determined by comparing the amount in each digest with that of a mock (no enzymes added) digest using a ΔCT method. Download the EpiTect Methyl PCR Array Excel-based data analysis template (www.sabiosciences.com/dna_methylation_data_analysis.php). Then, paste in the CT value data and analyze the automatically generated results by following the directions in the ‘Instructions’ worksheet of the Excel file.

#### Step 1: screening of candidate genes and testing of differential methylation status by qPCR of selected genes in a larger sample

Twenty-one human candidate genes (*ESR1, ESR2, AR, PGR, ESRRG, PPARA, PPARG, THRB, CYP1A1, CYP1B1, CYP19A1, VEGFA, MAPK1, MAPK3, STAT3, LIF, NR1I2, LAMP, TFF1, TNFα, and IL-4*) whose methylation at CpG islands (CGI)s may be related to phthalates based on a toxicogenomic database were selected [11,29]. The selection was based on the following criteria: (i) report to be related with DEHP in human studies and (ii) report to have an association with asthma. Figure 1 shows the study flow chart for selecting candidate genes. Initially, we analyzed DNA samples of 11 age and gender-matched children with the highest 5OH-MEHP levels in the cohort (high exposure group, 5 with and 6 without asthma) and 11 age and gender-matched children of the lowest 5OH-MEHP levels (low exposure group, 5 with and 6 without asthma). Twenty-two blood samples from 256 cohort children were screened by EpiTect Methyl II qPCR to assess the differential methylation between high and low exposure group (Table 1, Additional file 1: Table S2). The qPCR method is based on the detection of remaining input DNA after cleavage with a methylation-dependent restriction enzyme (Additional file 1: Figure S5) [30]. For those genes that did not have commercial kits for qPCR, we checked methylation status by pyrosequencing method via QIAGEN PyroMark Q24 instrument (QIAGEN, Inc., Valencia, CA, USA) [31].

Candidate genes with differential methylation were further tested in a larger sample of 256 cohort children. Then, we functionally validated candidate gene expression by measuring protein levels using ELISA. The association between candidate genes methylation status, exposure levels, and asthma was evaluated.

#### Step 2: replicating the phthalate-methylation association in a clinical sample

To investigate whether we can replicate the association between phthalate exposure and the identified gene methylation, we enrolled another sample of 54 children, who were presented by their parents in the clinic because of DEHP contaminated food events whose urine and blood samples were available.

#### Step 3: testing the association between gene methylation and asthma in children from CEAS and IOW birth cohort

A whole population birth cohort was established in the Isle of Wight, UK, in 1989 to prospectively study the natural history and etiology of asthma, eczema, and allergic conditions. A total of 1,456 children were followed up at 1, 2, 4, 10, and 18 years, as described elsewhere [15]. Blood or saliva samples were collected at the ages of 10 and 18 years for genetic analysis.

In the IOW cohort, DNA was measured using Illumina Infinium HumanMethylation450 BeadChips (Illumina, Inc., San Diego, CA, USA), as previously described [15]. The *TNFα* SNPs rs1800610 (an intron variant) was determined using Golden Gate Genotyping Assays (Illumina, Inc., San Diego, CA, USA) on the Bead Xpress Veracode platform (Illumina, Inc., San Diego, CA, USA). In brief, samples were fragmented and hybridized to the pool of allele-specific primer sets. Following an extension/ligation reaction, the samples were then hybridized to the Veracode bead pool and processed on the BeadXpress reader. Data were analyzed using the genotyping module of the GenomeStudio software package (Illumina, Inc., San Diego, CA, USA). The quality threshold for allele determination was set at a GenCall score 0.25 (scores #0.25 were ‘no calls’) with 98.3% and retained for further analysis.

### Statistical analysis

For statistical purposes, the concentration of 5OH-MEHP was log-transformed and also categorized into three exposure groups. Independent *t*-tests were performed to assess differences of methylation percentage (Met%: the percentage of methylated cytosine in the CGIs) between high and low exposure groups. The relationships between phthalate metabolite levels and *TNFα* promoter Met% and between Met% and *TNFα* protein expression levels were evaluated by linear regression. To evaluate the association of methylation status with asthma, we used logistic regression analyses and provided odds ratios and their 94% confidence limits. Selection of the confounders that were left in the model was based on the literature and the 10% rule of thumb of keeping a potential confounder in a model when removal changes the association of exposure and outcomes by more than 10%. All tests assumed a two-sided alternative hypothesis and a 0.05 significance level. All analyses were conducted using SAS software version 9.1 (SAS Institute, Cary, NC, USA).

In the IOW birth cohort, after cleaning the DNA-M data, beta (*β*) values are presented as the proportion of intensity of methylated (M) over the sum of methylated (M) and unmethylated (U) sites (*β* = M/[c + M + U] with *c* being a constant to prevent dividing by zero) were used to estimate the effect of DNA methylation [32]. The EpiTect Methyl II qPCR to measure methylation of *TNFα* (Chr6: 31543344–31544344) includes 11 CpG sites, of which 8 CpGs were also measured Illumina Infinium HumanMethylation450 BeadChip (Illumina, Inc., San Diego, CA, USA). Two of these did not pass quality control, three were disqualified due to probe SNPs, and three could be taken into consideration. Since we were comparing a Taiwanese sample with a sample from the UK, we took genetic difference into account. To this end, we estimated the statistical interaction between the *TNFα* SNPs rs1800610 and methylation level of cg10717214, cg04425624, and cg10650821 on the risk for asthma at age 18 years. In log-linear models, we estimated the interaction on a multiplicative scale. Statistically, we controlled for sex of the child, birth order, and duration of breastfeeding (weeks).

To evaluate how much risk of asthma in relation to exposure to phthalate is explained by changes in DNA methylation in TNFα gene, we conducted a mediation analysis [33]. The mediation analyses incorporated the different regression coefficients (exposure: 5OH-MEHP, mediator: *TNFα* 5′CGI, and outcome: asthma). The mediation proportions, the percentage change of the regression coefficients when we include an intermediate variable in the model, were calculated (Additional file 1: Figure S4). Both logistic and linear regressions were used.

## Additional file

Additional file 1: Supplementary information.
**Table S1.** Three candidate genes with differential methylation between low and high DEHP exposure in the screening. **Table S2.** The other 18 candidate genes without a significant differential methylation between low and high DEHP exposure in the screening (N = 22). **Figure S1.** The association between phthalate metabolites (5OH-MEHP) and *TNFα* DNA methylation percentage (Met%) in a larger sample of 256 children. **Figure S2.** The association of *TNF*α methylation percentage (Met%) and TNFα protein level. **Figure S3.** Risk ratio of asthma at age 18 years versus percent methylation of cg10717214 at different genotypes of *TNFα* SNP rs1800610. **Figure S4**. Details on the mediation analysis approach. **Figure S5.** The workflow chart outlining the identification of the differentially methylated genes in a step-by-step manner by qPCR.

## Abbreviations

5OH-MEHP: mono(2-ethyl-5-hydroxyhexyl)phthalate
AR: Androgen receptor
CGI: CpG island
DEHP: Di-(2-ethylhexyl) phthalate
IL-4: Interleukin 4
*TNFα*: Tumor necrosis factor-alpha
