# Supplementary material for: Effects of phthalate exposure on asthma may be mediated through alterations in DNA methylation
Source: Clin Epigenetics. 2015 Mar 15;7(1):27. doi: 10.1186/s13148-015-0060-x (PMC4424541; doi:10.1186/s13148-015-0060-x)
Supplement: Additional file 1: Supplementary information. — Table S1. Three candidate genes with differential methylation between low and high DEHP exposure in the screening. Table S2. The other 18 candidate genes without a significant differential methylation between low and high DEHP exposure in the screening (N = 22). Figure S1. The association between phthalate metabolites (5OH-MEHP) and TNFα DNA methylation percentage (Met%) in a larger sample of 256 children. Figure S2. The association of TNFα methylation percentage (Met%) and TNFα protein level. Figure S3. Risk ratio of asthma at age 18 years versus percent methylation of cg10717214 at different genotypes of TNFα SNP rs1800610. Figure S4. Details on the mediation analysis approach. Figure S5. The workflow chart outlining the identification of the differentially methylated genes in a step-by-step manner by qPCR. [file 13148_2015_60_MOESM1_ESM.doc]

**Supplement**

**Table S1.** Three candidate genes with differential methylation between low and high DEHP exposure in the screening

|  | Gene ID | | Scatter plot of Met% between low and high exposure | |
| --- | --- | --- | --- | --- |
|  |  |  |  |  |
|  |  |  | | 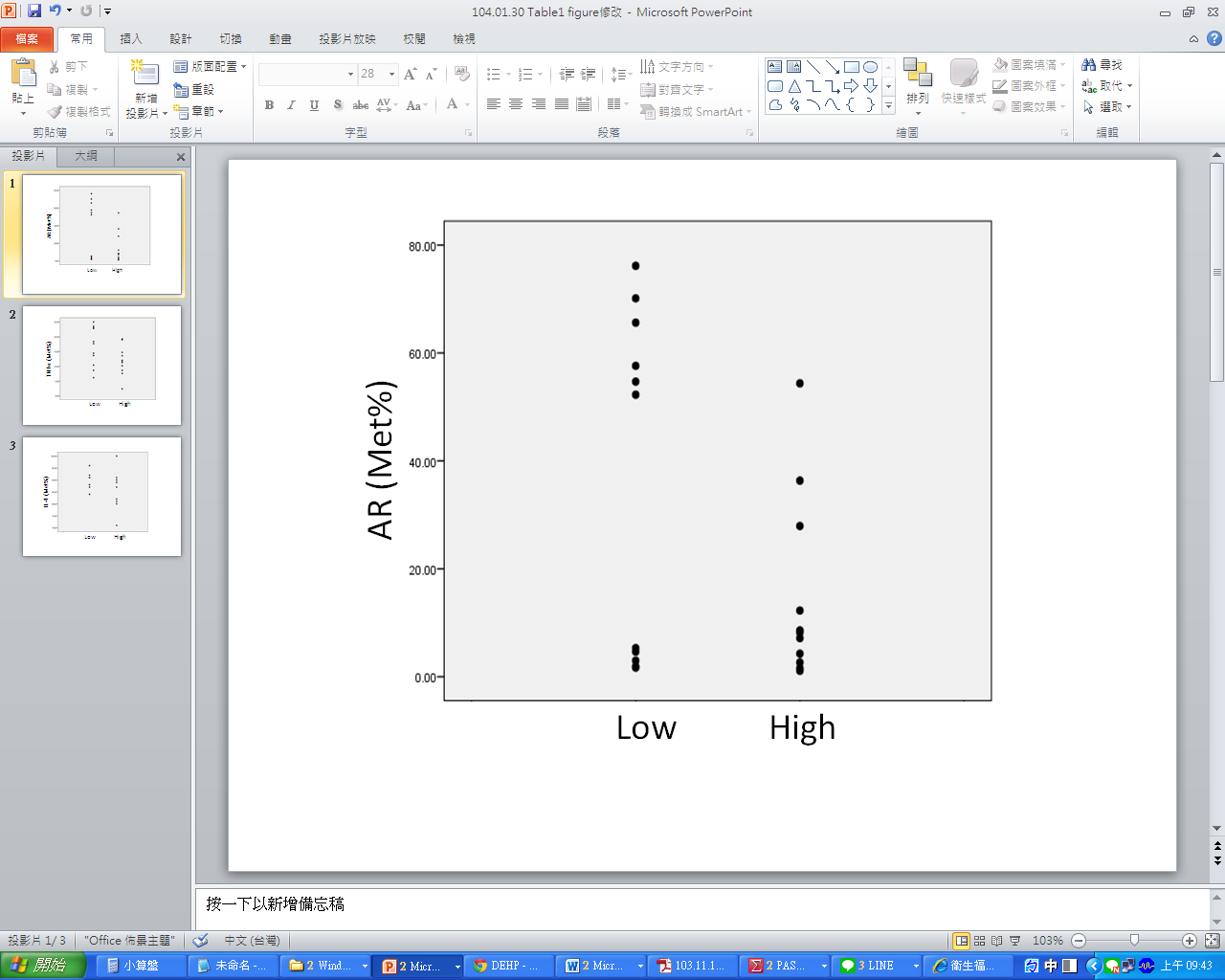 |
|  | AR (androgen receptor) (367) |  |  |  |
|  |  |  |  | 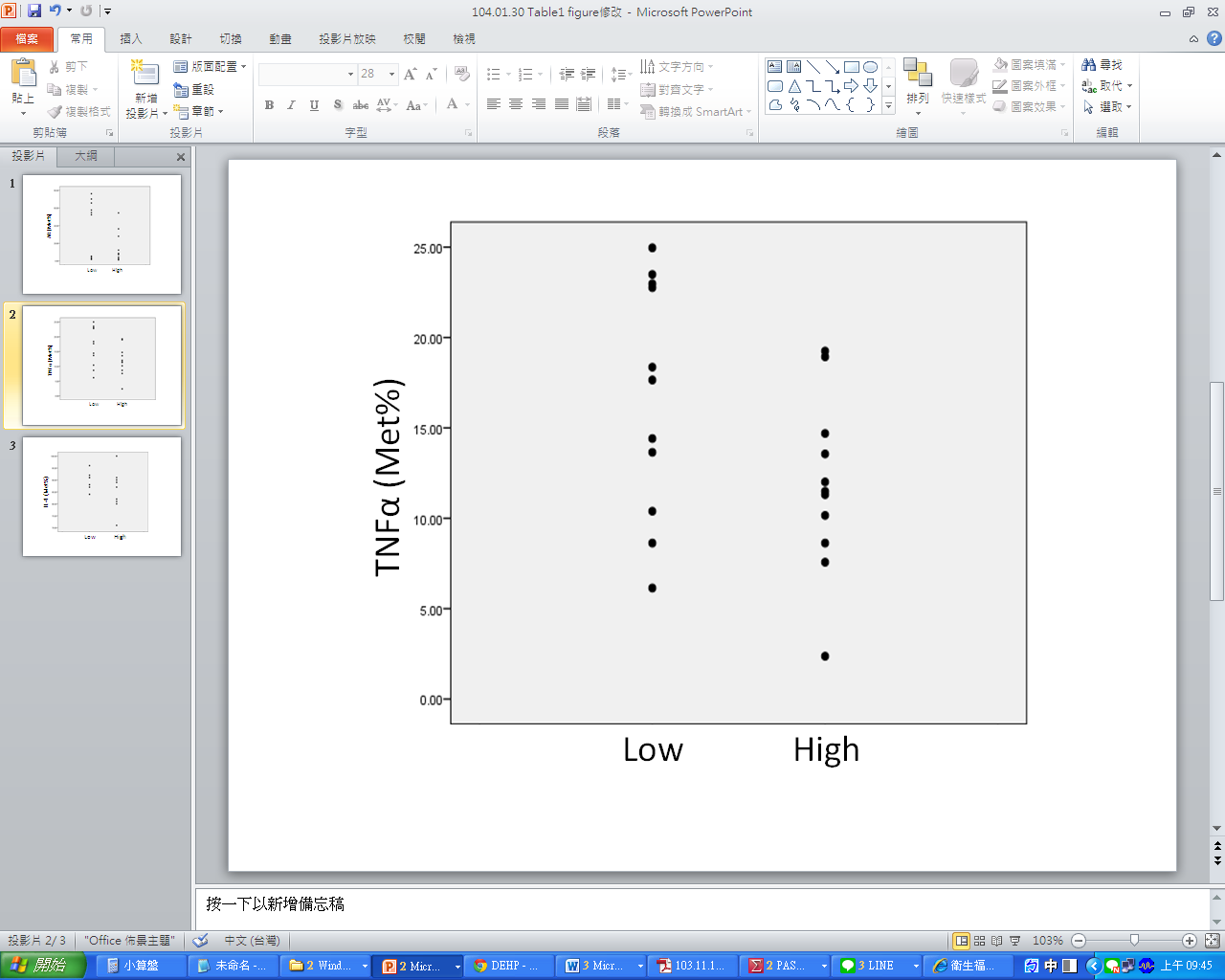 |
|  | TNFα (tumor necrosis  factor α) 7124) |  |  |  |
|  |  |  |  | 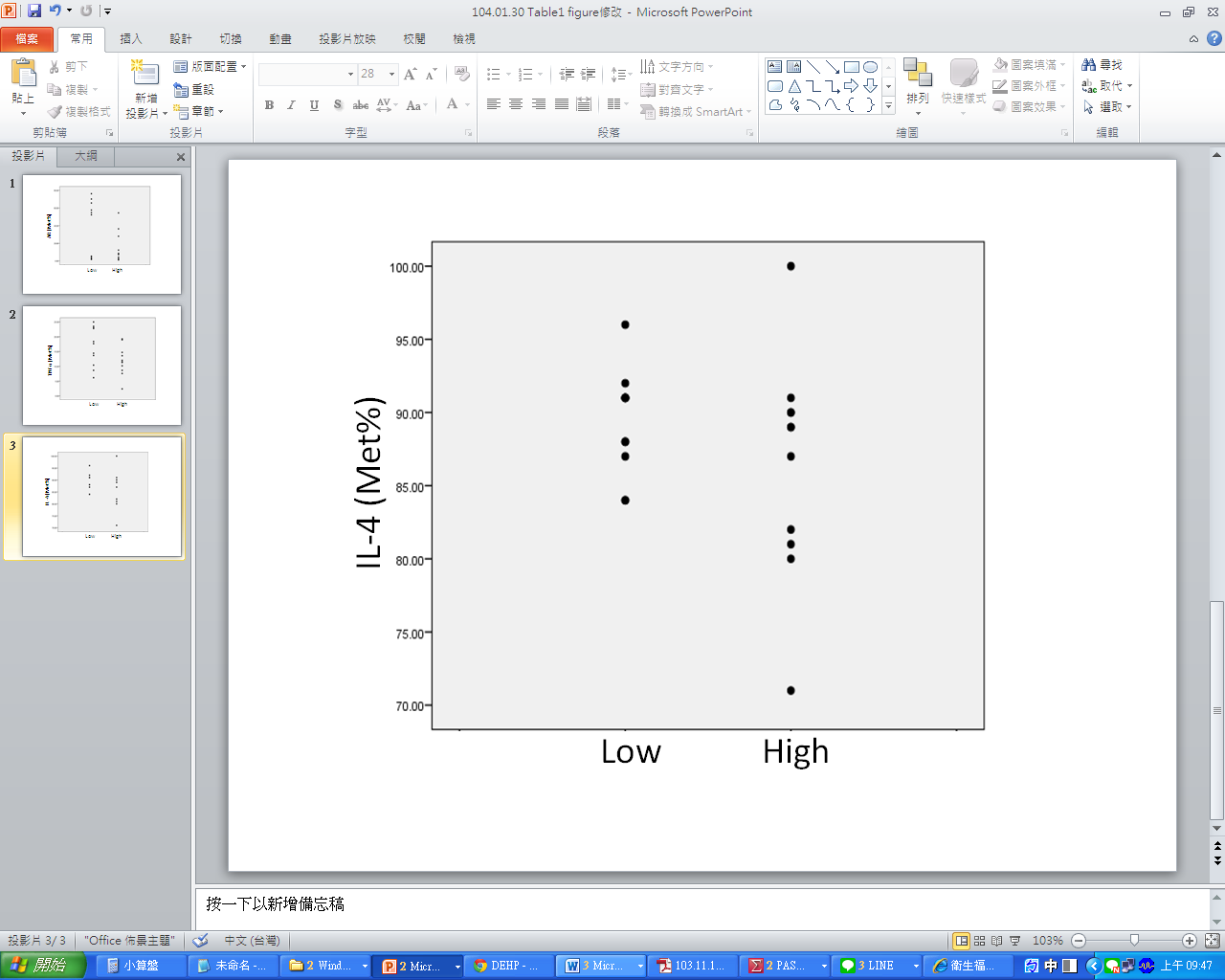 |
|  | IL-4 (Interleukin-4) (16189) |  |  |  |
|  |  |  |  |  |
|  |  |  | |  |

[**Table**](http://www.ncbi.nlm.nih.gov/pmc/articles/PMC2637989/table/pone-0004488-t002/) **S2.** The other 18 candidate genes without a significant differential methylation between low and high DEHP exposure in the screening (N=22).

| Gene ID | CpG island location | Assay details^b^ | Scatter plot of Met% between low and high exposure |
| --- | --- | --- | --- |
|  | TSS position | Met%^a^(mean ± SD) of low and high DEHP exposure |  |
| **ESR1**  **(Estrogen receptor 1)**  **(2099)** | Chr6:  152128822 - 152129771 | Catalog no:335002EPHS112843-1A  CpG island location:chr6:152128822-152129771  Assay position(central point): chr6:152129153  PCR product size:233-bp  NCBI build no:37 | 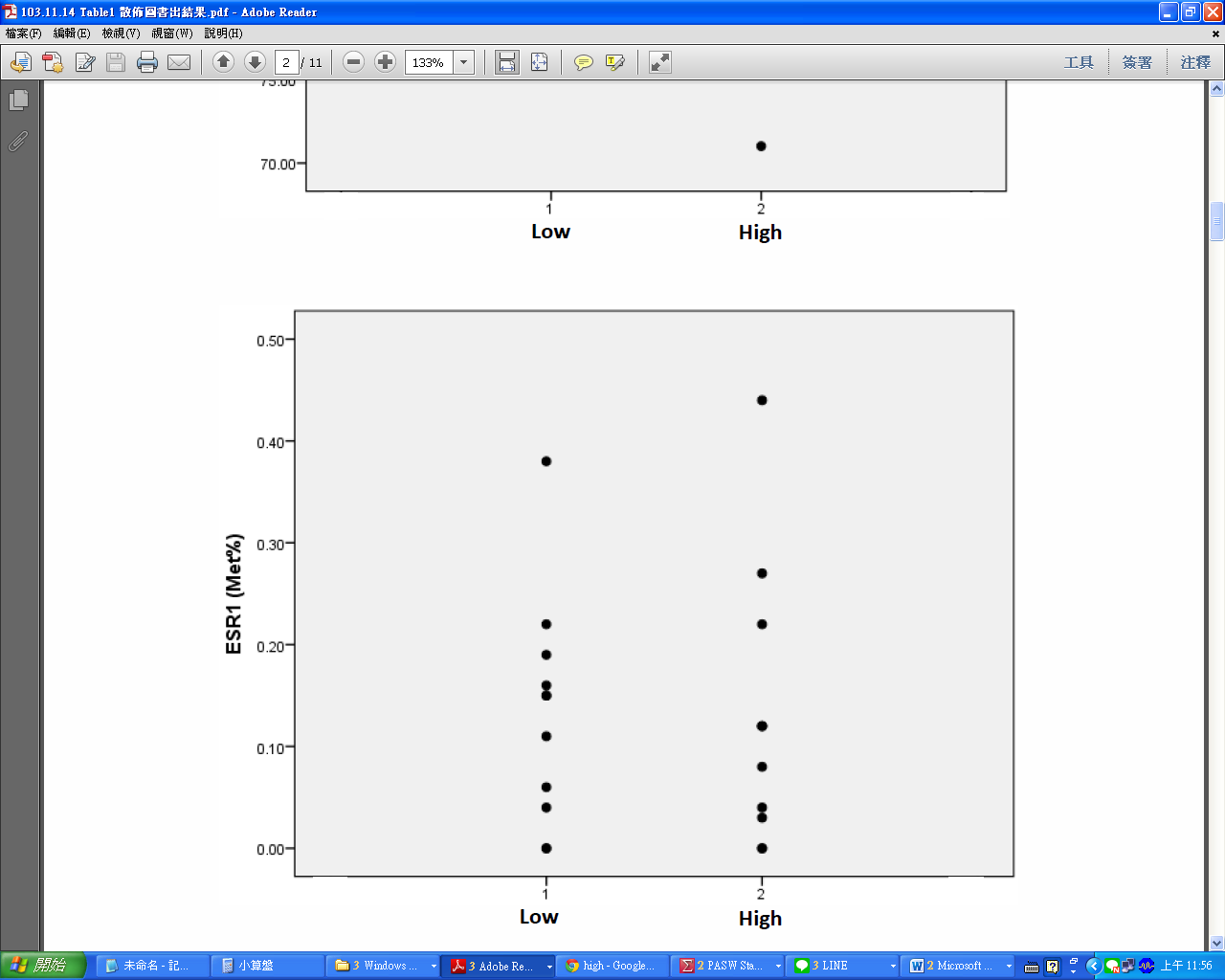 |
|  | TSS:152128813 | Low vs. high exposure  0.13±0.11 vs. 0.13±0.13  p=0.973 |  |
| **ESR2**  **(Estrogen receptor 2)**  **(2100)** | Chr14:  64805109 - 64805785 | Catalog no:335002EPHS104140-1A  CpG island location:chr14:64805109-64805785  Assay position(central point): chr14:64805625  PCR product size:300-bp  NCBI build no:37 | 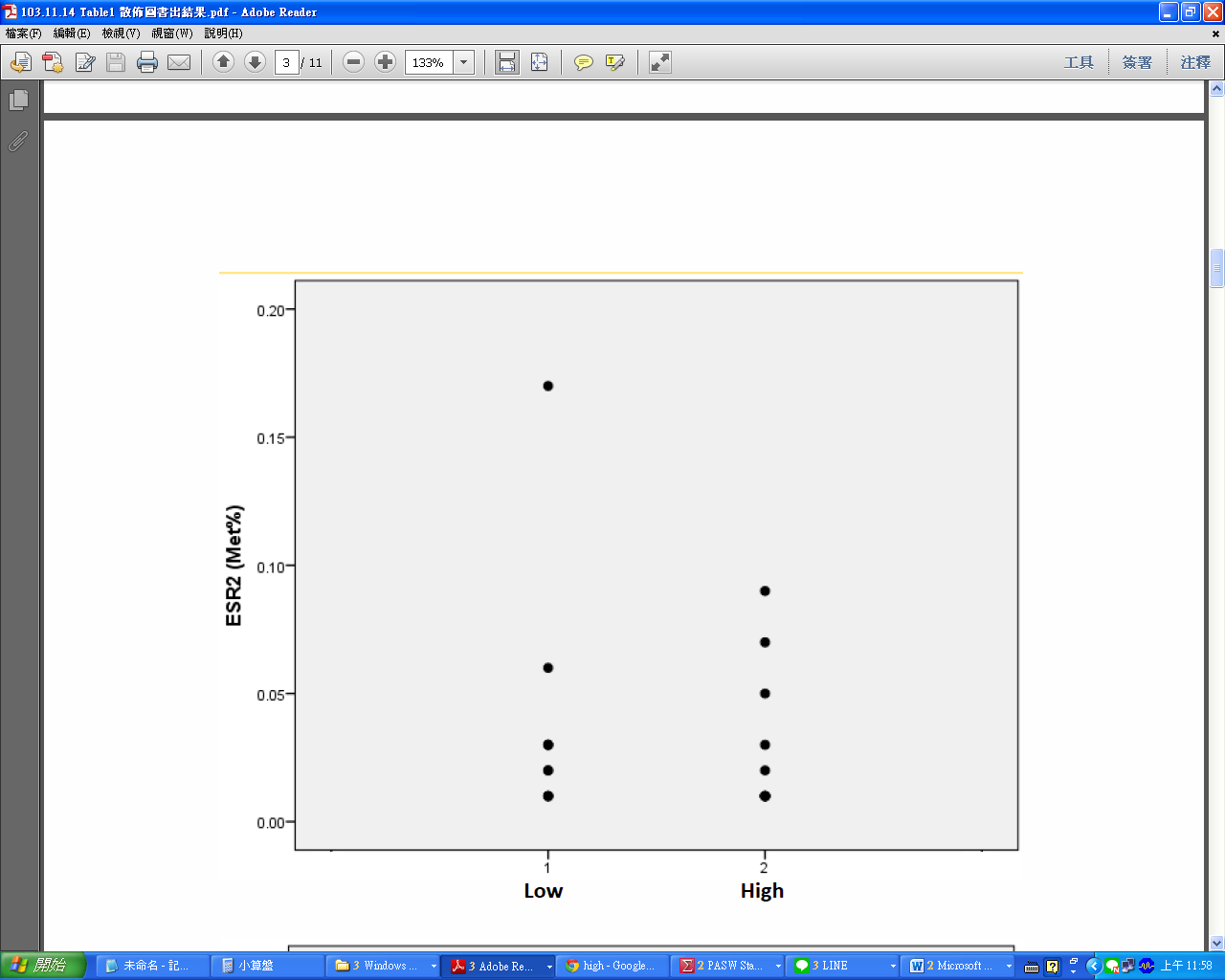 |
|  | TSS: 64805268 | Low vs. high exposure  0.04±0.05 vs. 0.03±0.03  p=0.664 |  |
| **PGR**  **(Progesterone receptor)**  **(5241)** | Chr11: 100999946 - 101000437 | Catalog no:335002EPHS102708-1A  CpG island location:chr11:100999946-101000437  Assay position(central point): chr11:101000191  PCR product size:298 -bp  NCBI build no:37 | 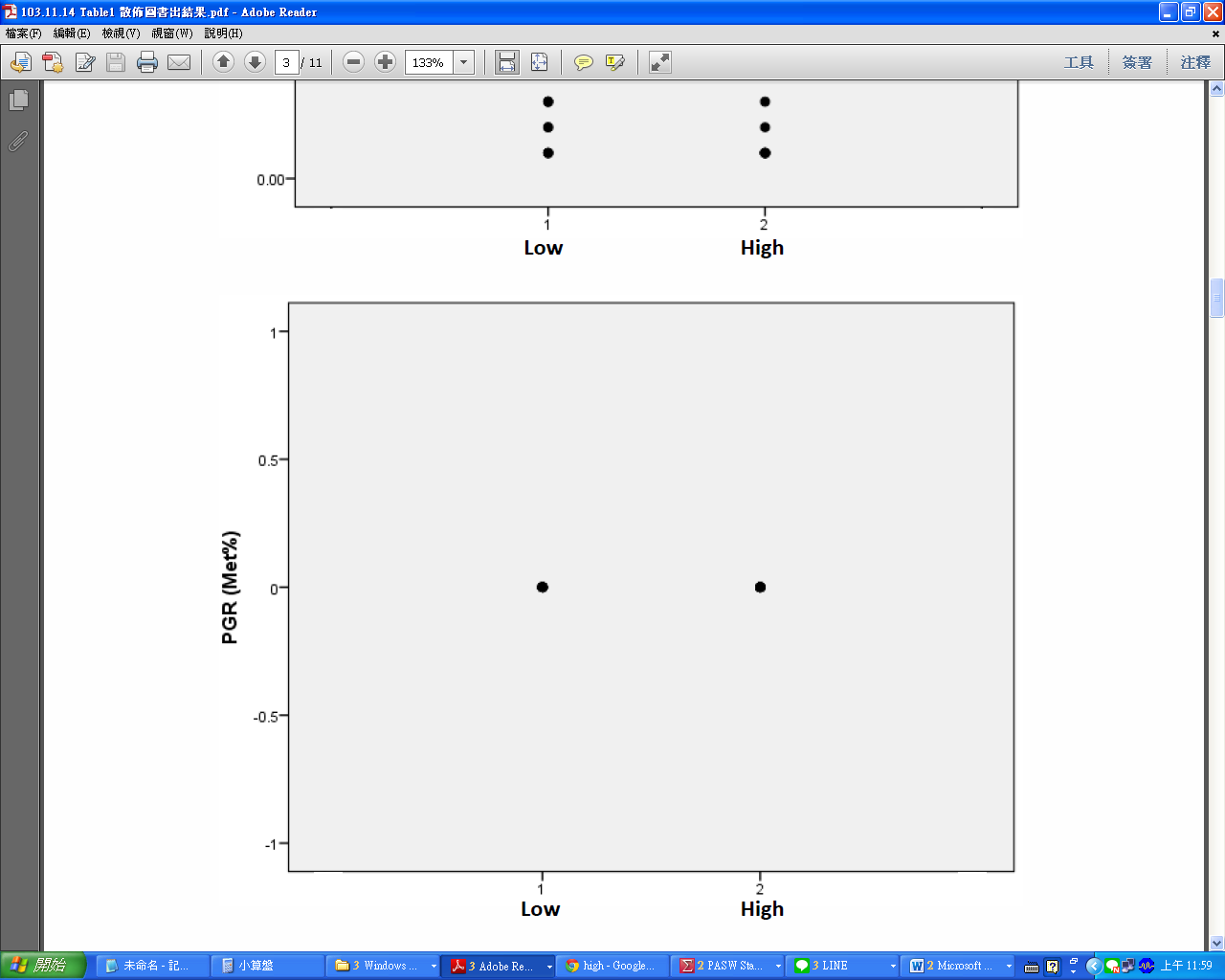 |
|  | TSS: 101000544 | Low vs. high exposure  0 vs. 0 |  |
| **ESRRG**  **(Estrogen-related receptor gamma)**  **(2104)** | Chr1:  217310749 - 217311178 | Catalog no:335002EPHS101274-1A  CpG island location:chr1:217310749 -217311178  Assay position(central point): chr1:217310980  PCR product size:172 -bp  NCBI build no:37 | 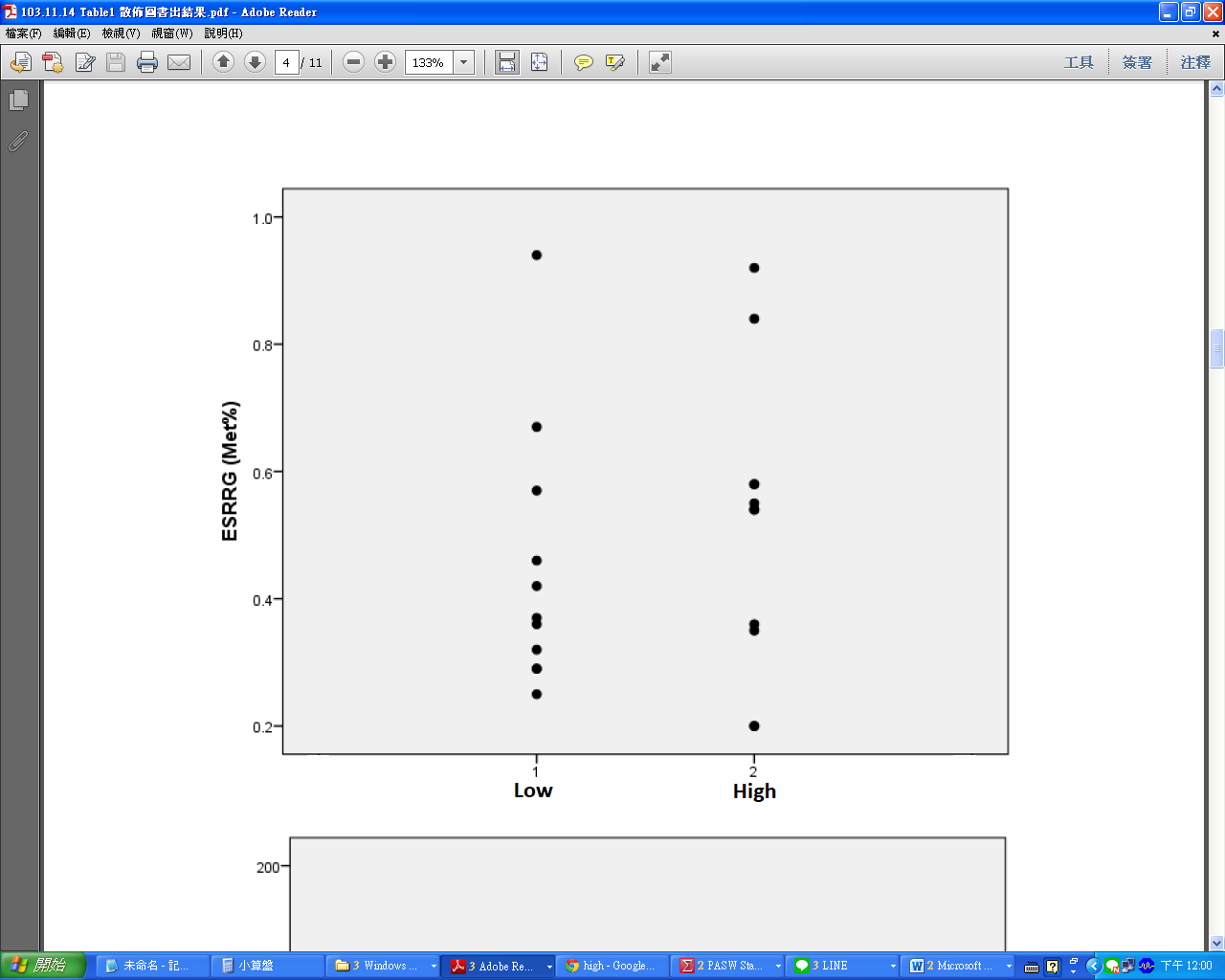 |
|  | TSS: 217311097 | Low vs. high exposure  0.45±0.21 vs. 0.52±0.23  p=0.491 |  |
| **PPARA**  **(Peroxisome proliferator-activated receptor alpha)**  **(5465)** | Chr22: 46545480 - 46547412 | Catalog no:335002EPHS109941-1A  CpG island location:chr22:46545480 -46547412  Assay position(central point): chr22:46546450  PCR product size:250 -bp  NCBI build no:37 | 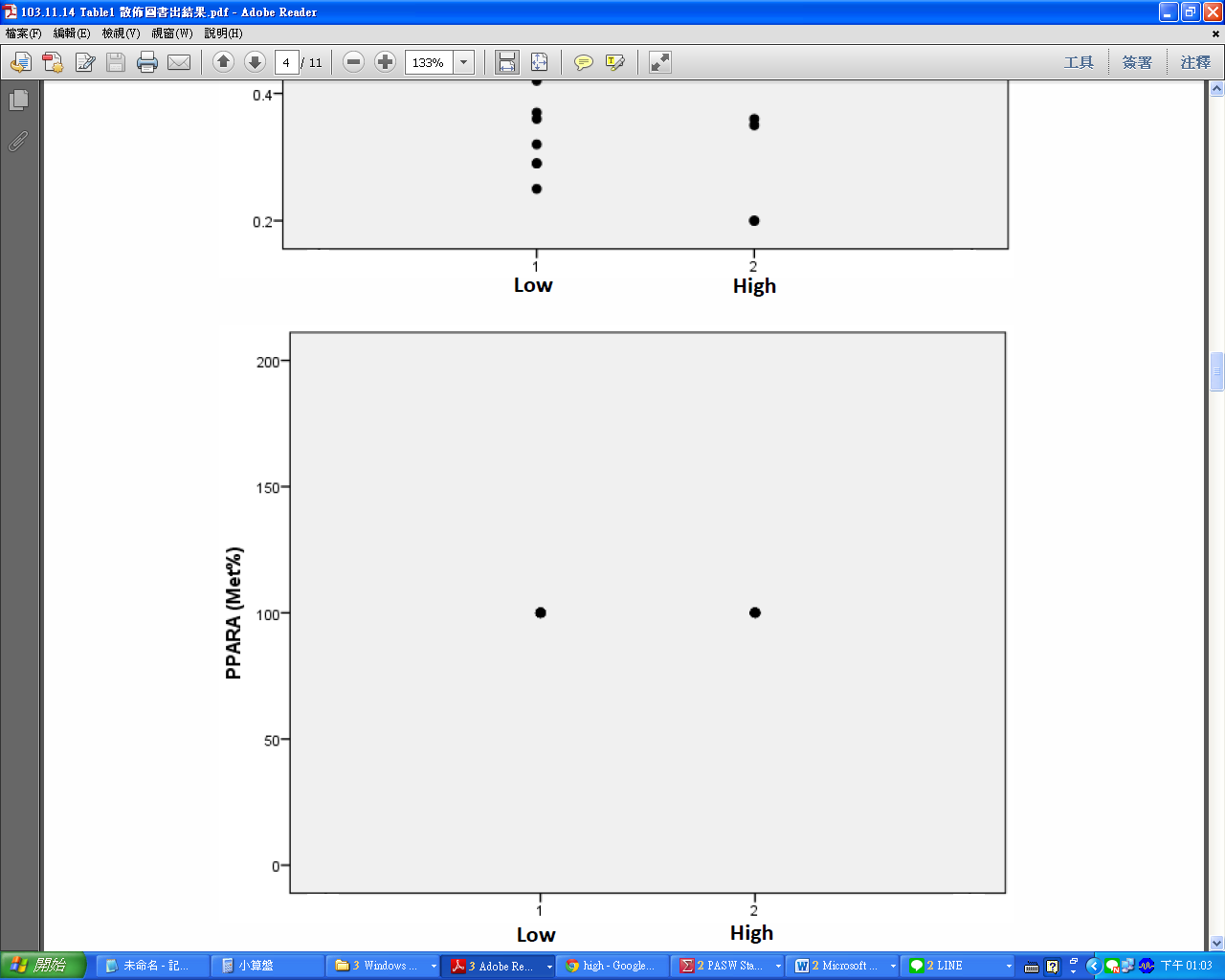 |
|  | TSS: 46546498 | Low vs. high exposure  100.0±0.0 vs. 100.0±0.0 |  |
| **PPARG**  **(Peroxisome proliferator-activated receptor gamma)**  **(5468)** | Chr3:  12329427 - 12330333 | Catalog no:335002EPHS110053-1A  CpG island location:chr3:12329427 -12330333  Assay position(central point): chr3:12329880  PCR product size:260 -bp  NCBI build no:37 | 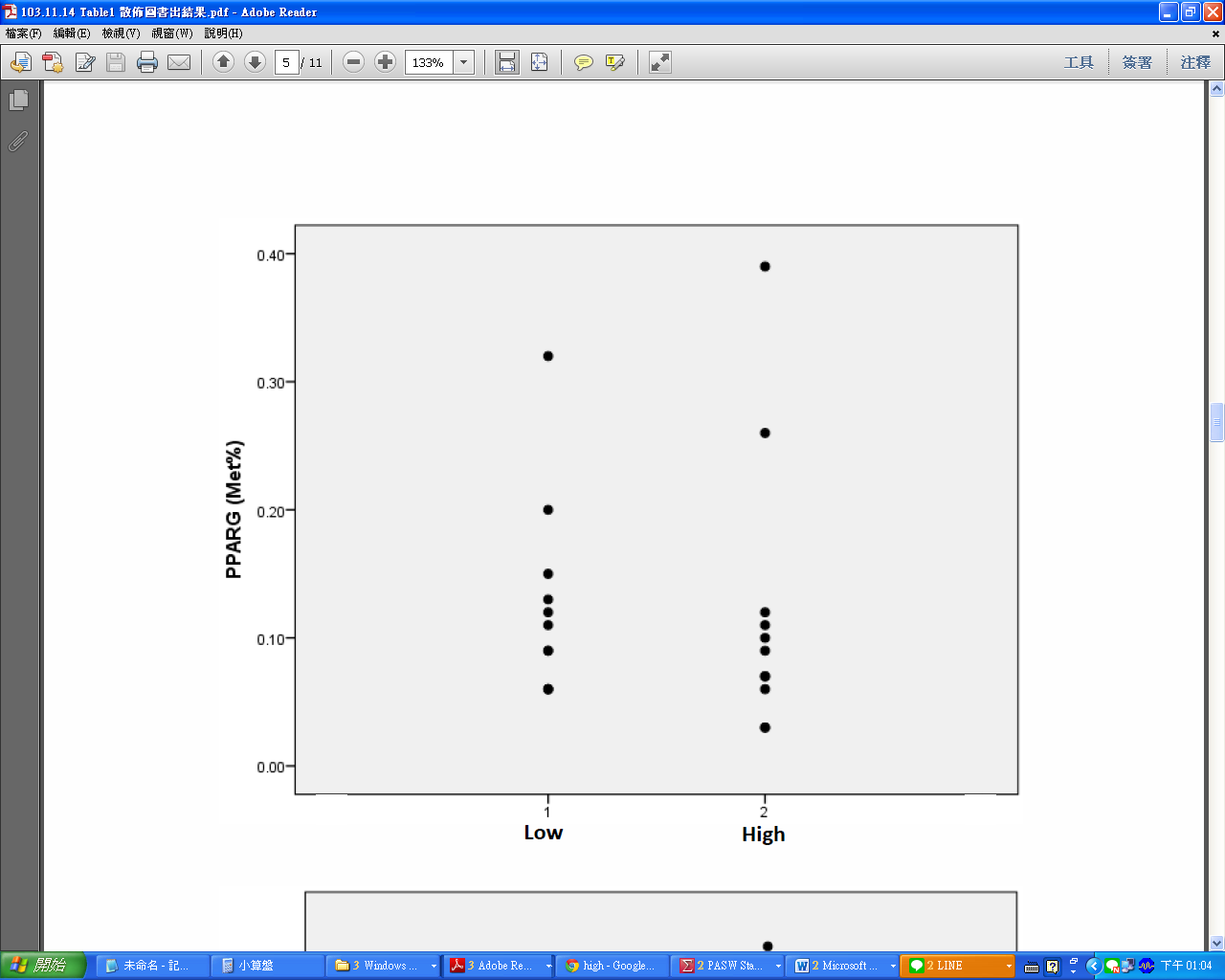 |
|  | TSS: 12329348 | Low vs. high exposure  0.13±0.08 vs. 0.12±0.11  p=0.894 |  |
| **THRB**  **(Thyroid hormone receptor beta)**  **(7068)** | Chr3: 24535844 - 24537436 | Catalog no:335002EPHS110100-1A  CpG island location:chr3:24535844-24537436  Assay position(central point): chr3:24536285  PCR product size:200 -bp  NCBI build no:37 | 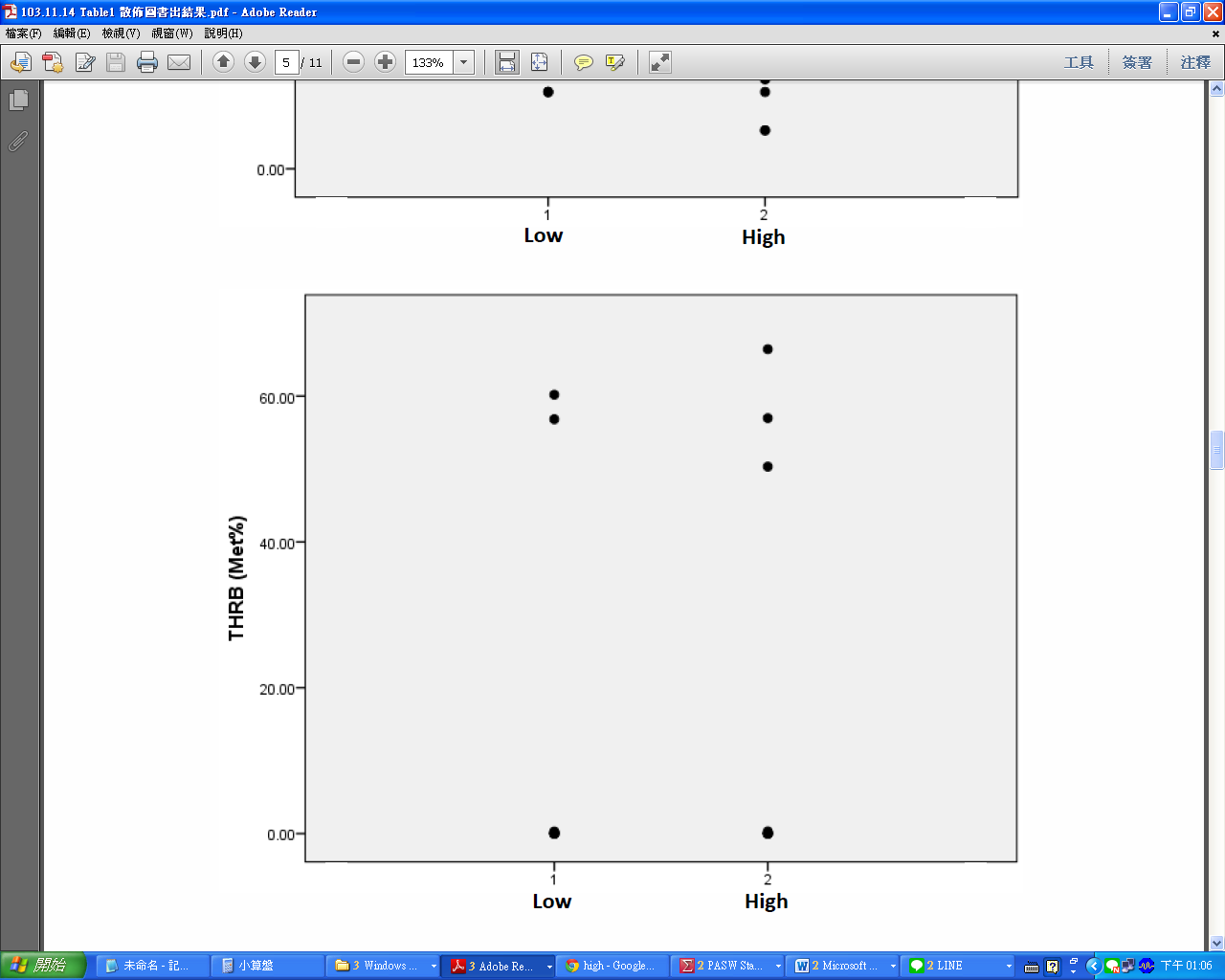 |
|  | TSS: 24536313 | Low vs. high exposure  10.69±23.66 vs. 15.84±27.27  p=0.642 |  |
| **CYP1A1**  **(Cytochrome P450, family 1, subfamily A, polypeptide 1)**  **(1543)** | Chr15: 75018186 - 75019336 | Catalog no:335002EPHS104685-1A  CpG island location:chr15:75018186-75019336  Assay position(central point): chr15:75018753  PCR product size:200 -bp  NCBI build no:37 | 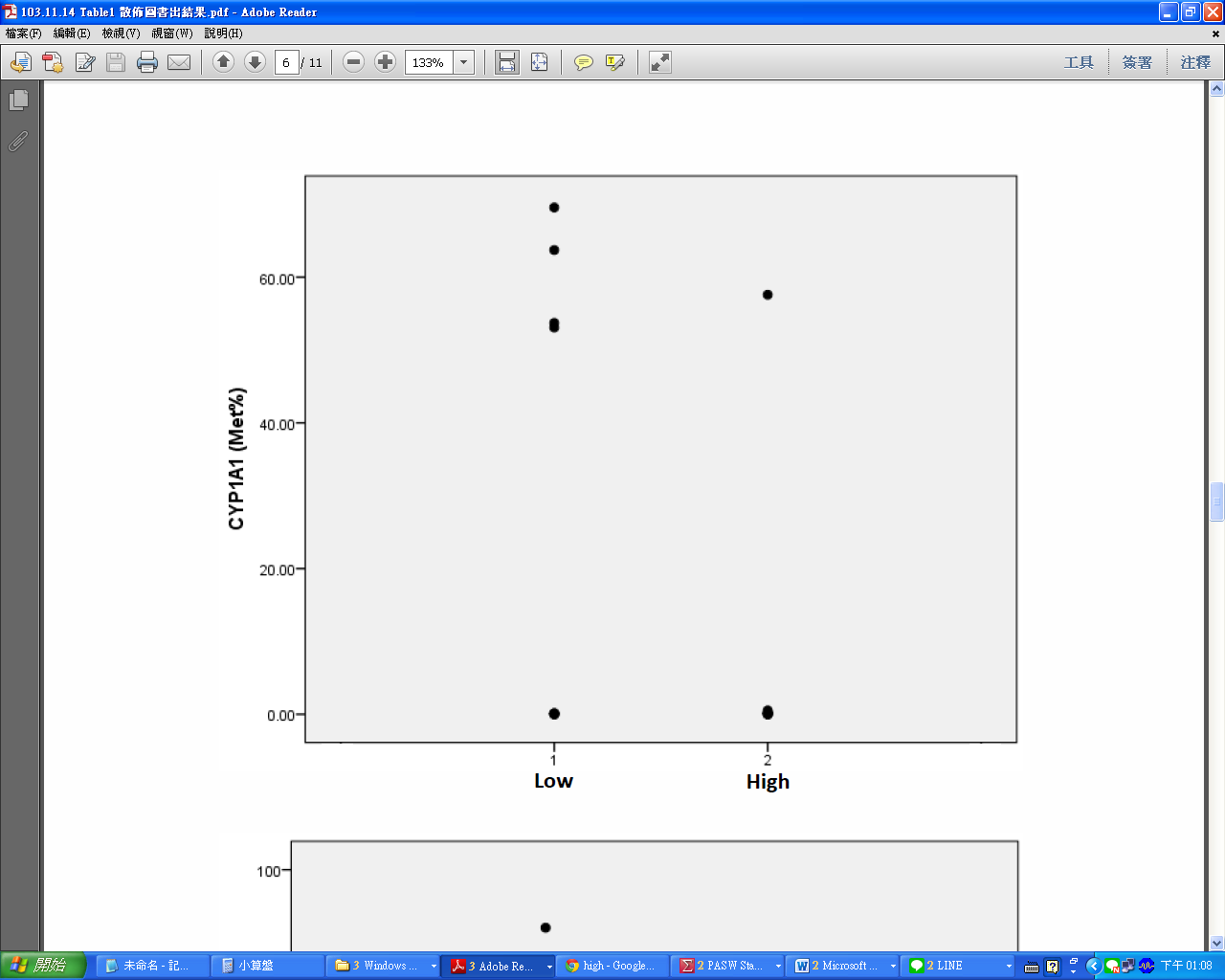 |
|  | TSS: 75017877 | Low vs. high exposure  21.87±30.56 vs. 5.37±17.32  p=0.139 |  |
| **CYP1B1**  **(Cytochrome P450, family 1, subfamily B, polypeptide 1)**  **(1545)** | Chr2:  38301276 - 38304518 | Catalog no:335002EPHS108174-1A  CpG island location:chr2:38301276-38304518  Assay position(central point): chr2:38302031  PCR product size:182-bp  NCBI build no:37 | 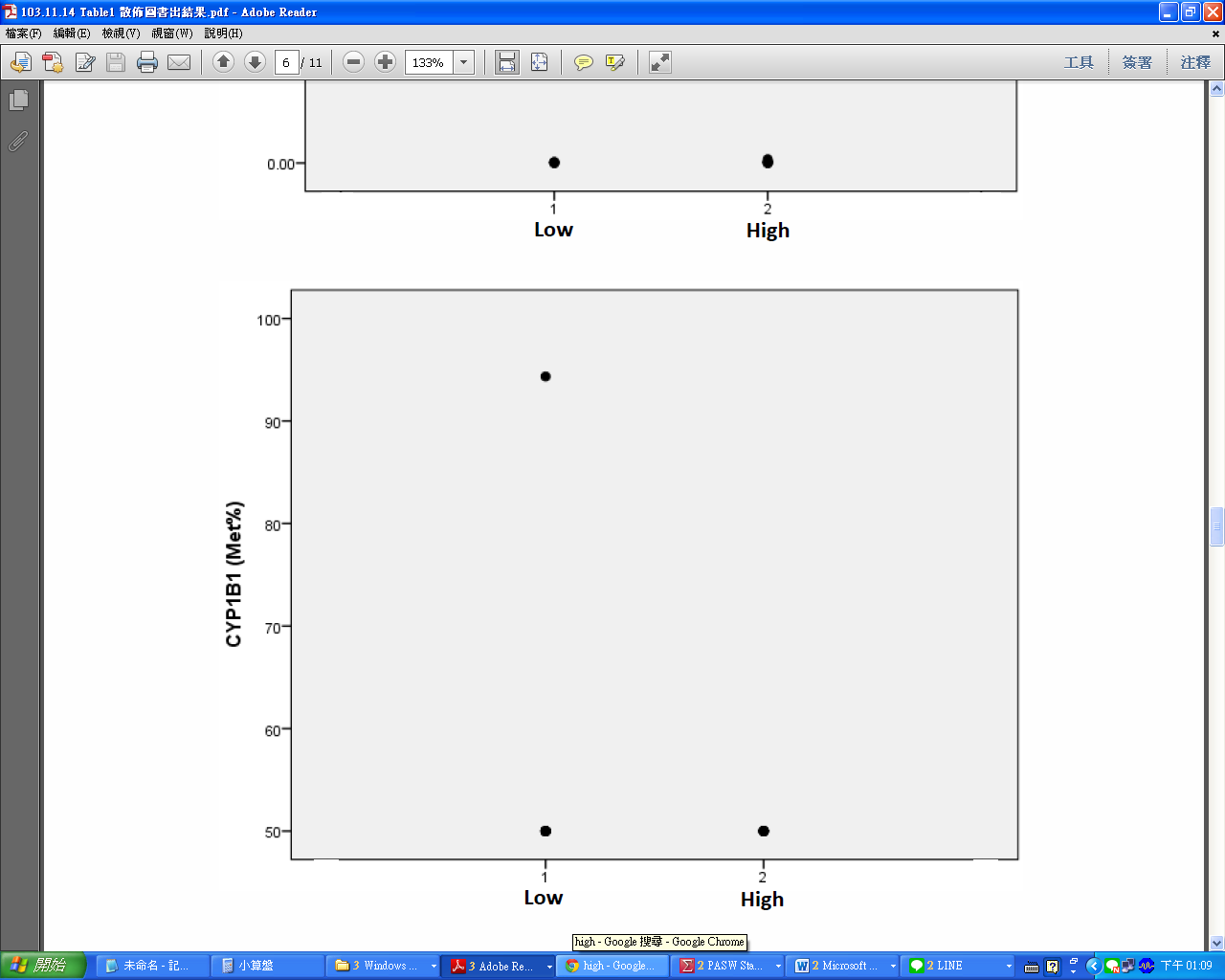 |
|  | TSS: 38303323 | Low vs. high exposure  54.03±13.37 vs. 50.00±0.00  p=0.341 |  |
| **CYP19A1**  **(Cytochrome P450, family 19, subfamily A, polypeptide 1)**  **(1588)** | Chr15: 51633724 - 51634318 | Catalog no:335002EPHS104541-1A  CpG island location:chr15:51633724-51634318  Assay position(central point): chr15: 51634021  PCR product size:250-bp  NCBI build no:37 | 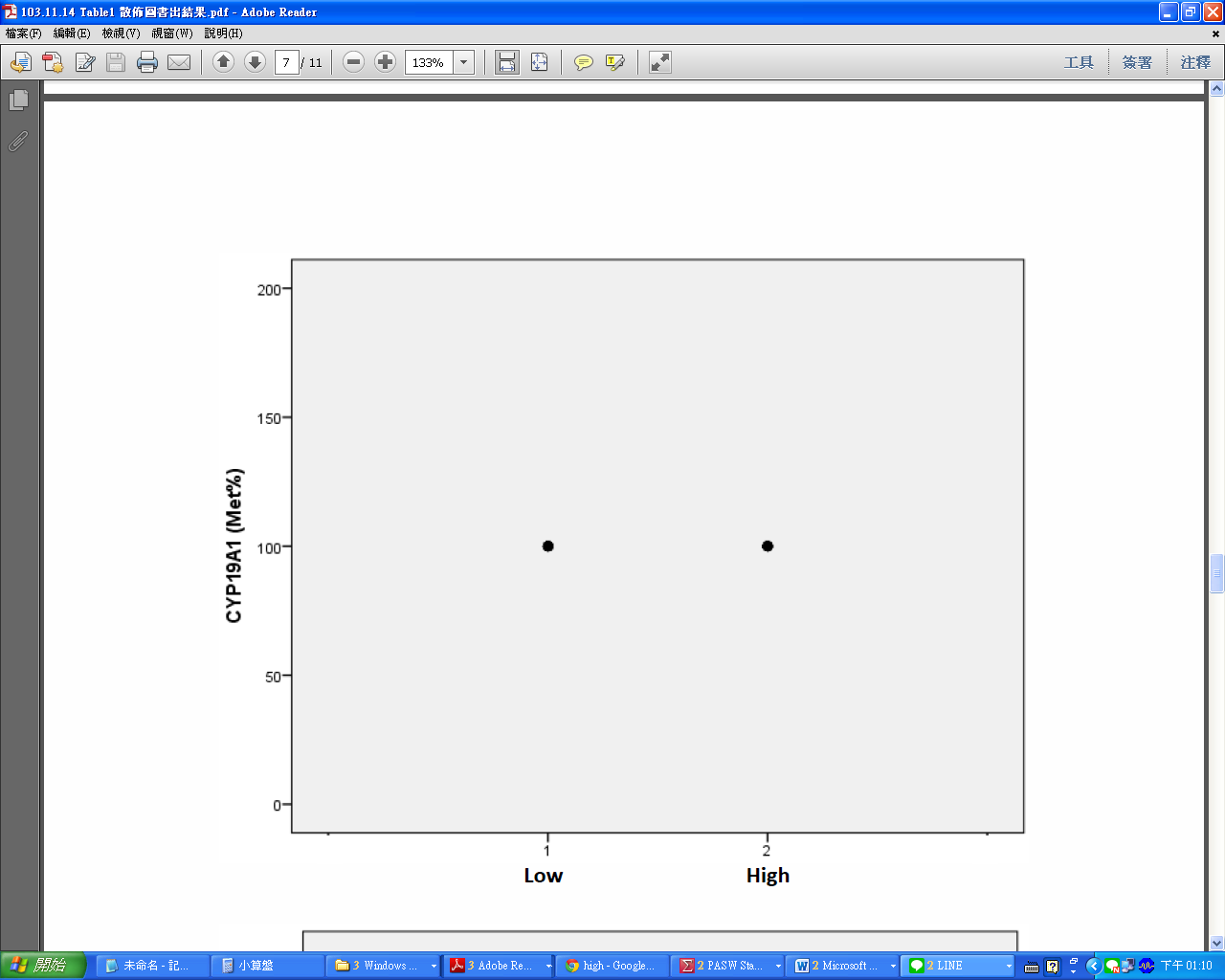 |
|  | TSS: 51630795 | Low vs. high exposure  100.00±0.00 vs. 100.00±0.00 |  |
| **MAPK3**  **(Mitogen-activated protein kinase 3)**  **(5595)** | Chr16:  30134220 - 30134488 | Catalog no:335002EPHS105199-1A  CpG island location:chr16:30134220-30134488  Assay position(central point): chr16:30134336  PCR product size:150-bp  NCBI build no:37 | 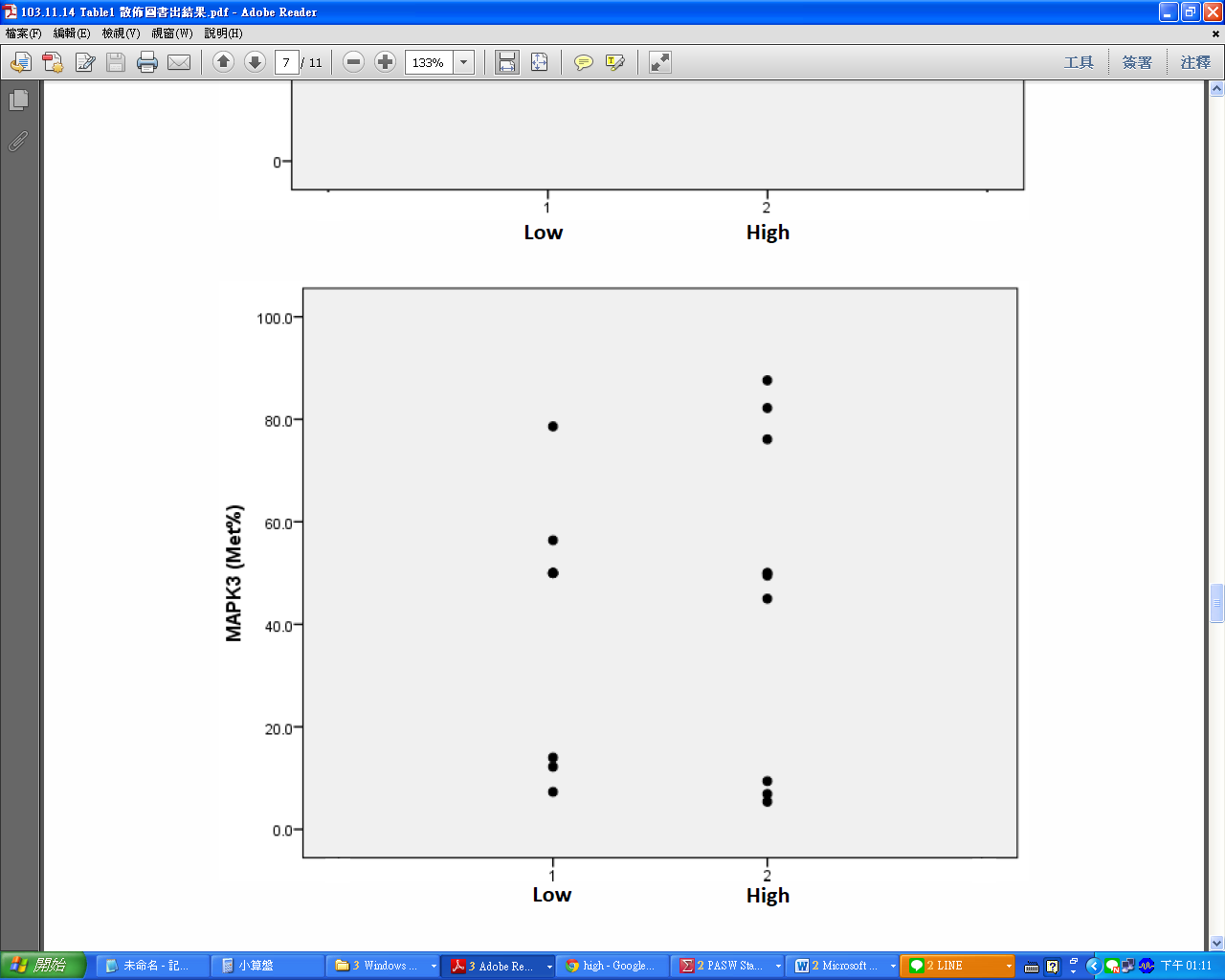 |
|  | TSS:30134630 | Low vs. high exposure  40.94±24.16 vs. 46.21±30.75  p=0.686 |  |
| **STAT3**  **(Signal transducer and activator of transcription 3)**  **(6774)** | Chr17:  40539837 - 40540775 | Catalog no:335002EPHS106011-1A  CpG island location:chr17:40539837-40540775  Assay position(central point): chr17:40540102  PCR product size:150-bp  NCBI build no:37 | 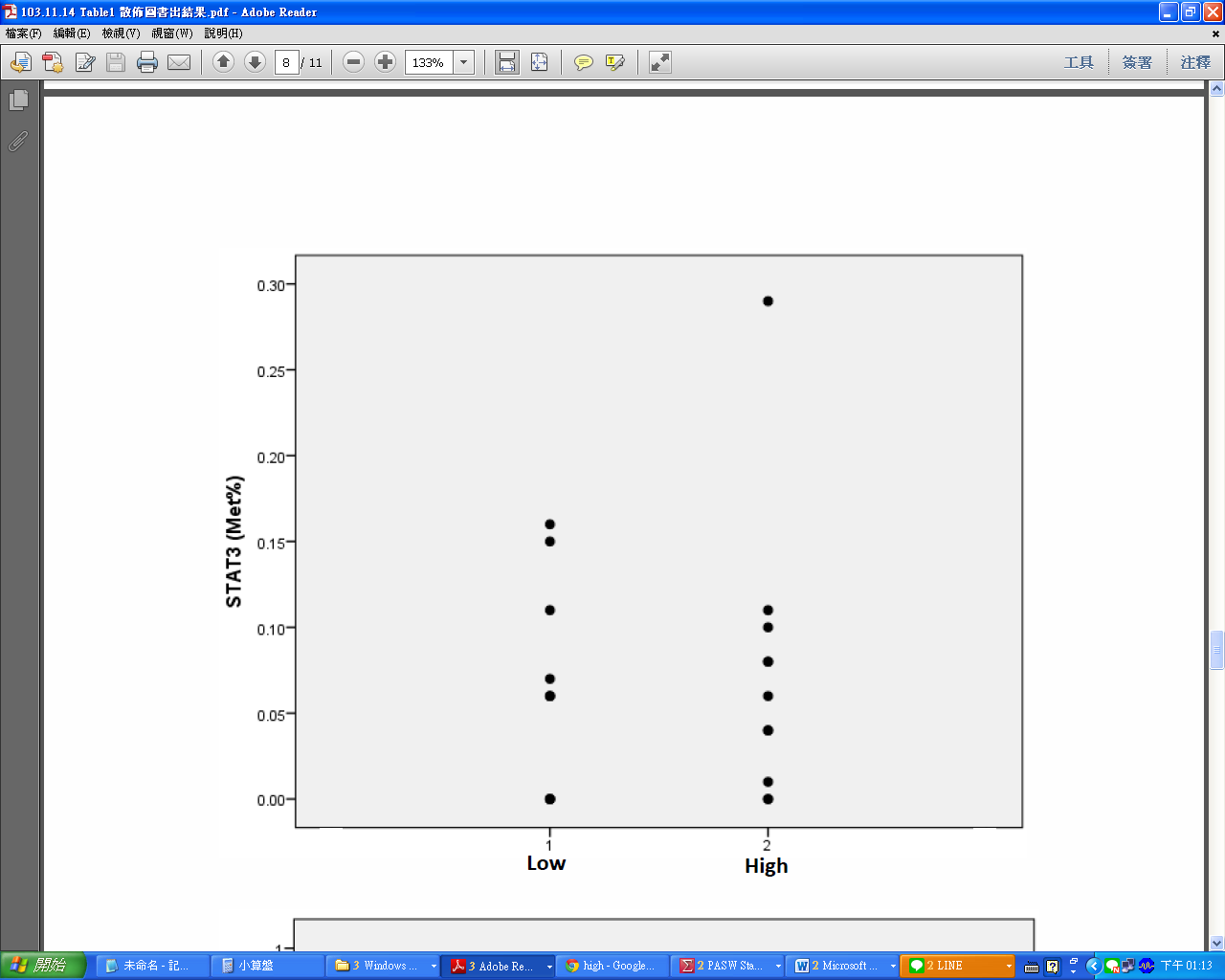 |
|  | TSS:40540513 | Low vs. high exposure  0.06±0.06 vs. 0.07±0.08  p=0.679 |  |
| **LIF**  **(leukemia inhibitory factor)**  **(3976)** | Chr22:  30642437 - 30642671 | Catalog no:335002EPHS109763-1A  CpG island location:chr22:30642437-30642671  Assay position(central point): chr22:30642542  PCR product size:150-bp  NCBI build no:37 | 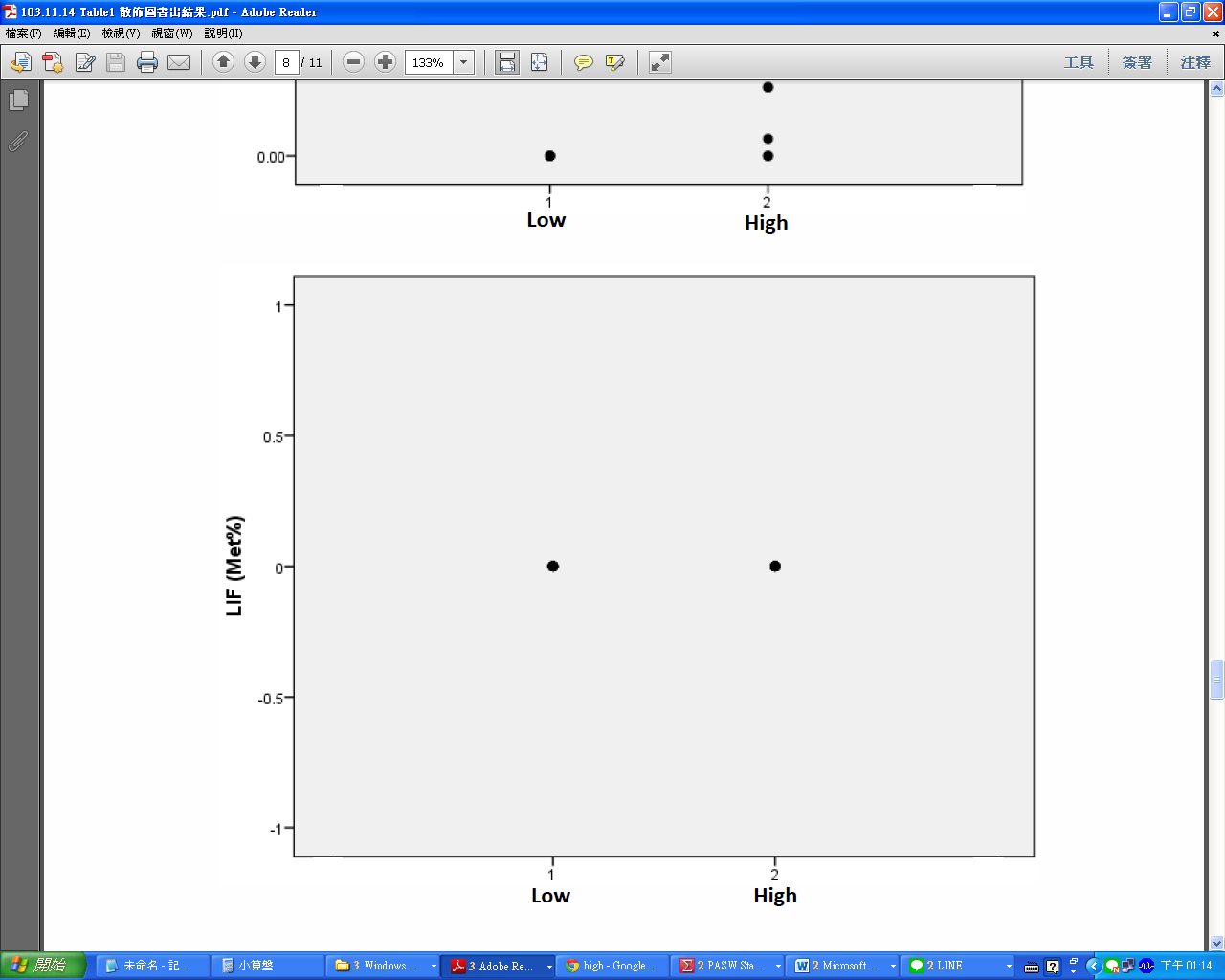 |
|  | TSS:30642796 | Low vs. high exposure  0 vs. 0 |  |
| **NR1L2**  **(Pregnane-X-receptor)**  **(8856)** | Chr3:  119528619 -119529338 | Catalog no:335002MePH80049-1A  CpG island location:chr3:119528619-119529338  Assay position(central point): chr3:119529021  PCR product size:215-bp  NCBI build no:37 | 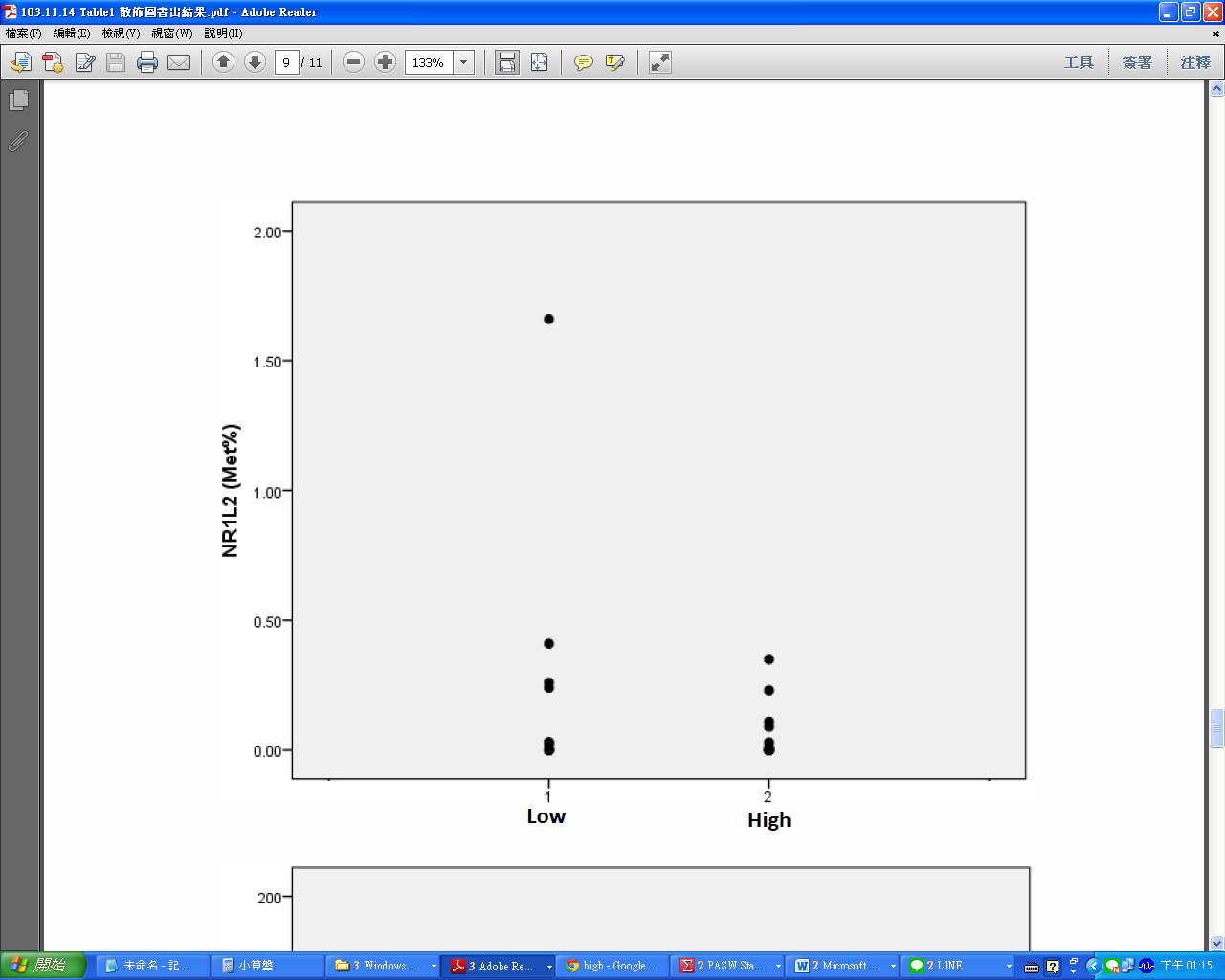 |
|  | TSS:119501557 | Low vs. high exposure  0.24±0.49 vs. 0.07±0.12  p=0.287 |  |
| **LAMP3**  **(lysosomal-associated membrane protein 3)**  **(27074)** | Chr3: 182880277 - 182880722 | Catalog no:335002EPHS110726-1A  CpG island location:chr3: 182880277-182880722  Assay position(central point): chr3: 182880499  PCR product size:250-bp  NCBI build no:37 | 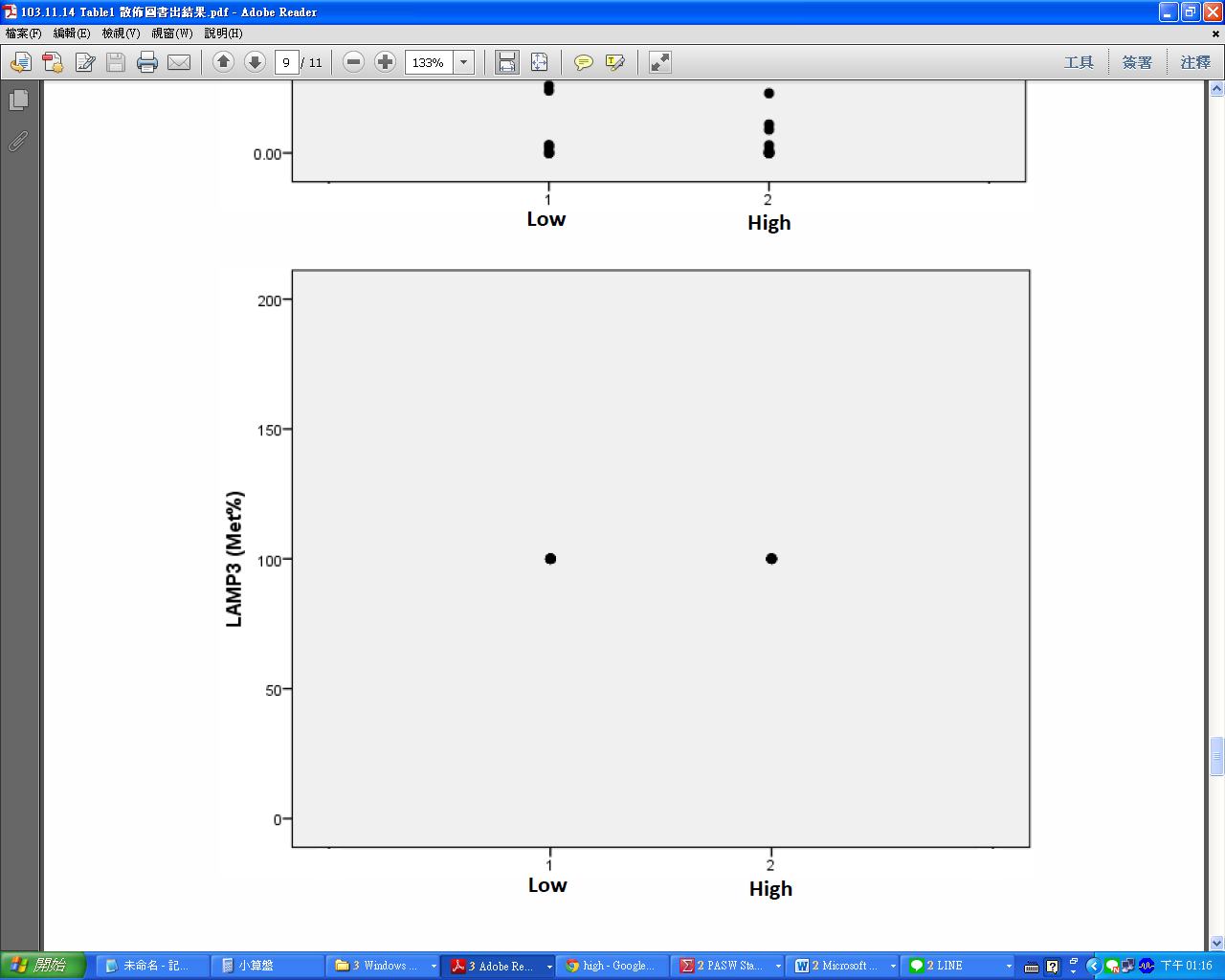 |
|  | TSS:182880667 | Low vs. high exposure  100.00±0.00 vs. 100.00±0.00 |  |
| **TFF1**  **(Trefoil factor 1)**  **(7031)** | Chr21:  43784646 - 43785645 | Catalog no:335002MePH80048-1A  CpG island location:chr21:43784646-43785645  Assay position(central point): chr21:43784887  PCR product size:280-bp  NCBI build no:37 | 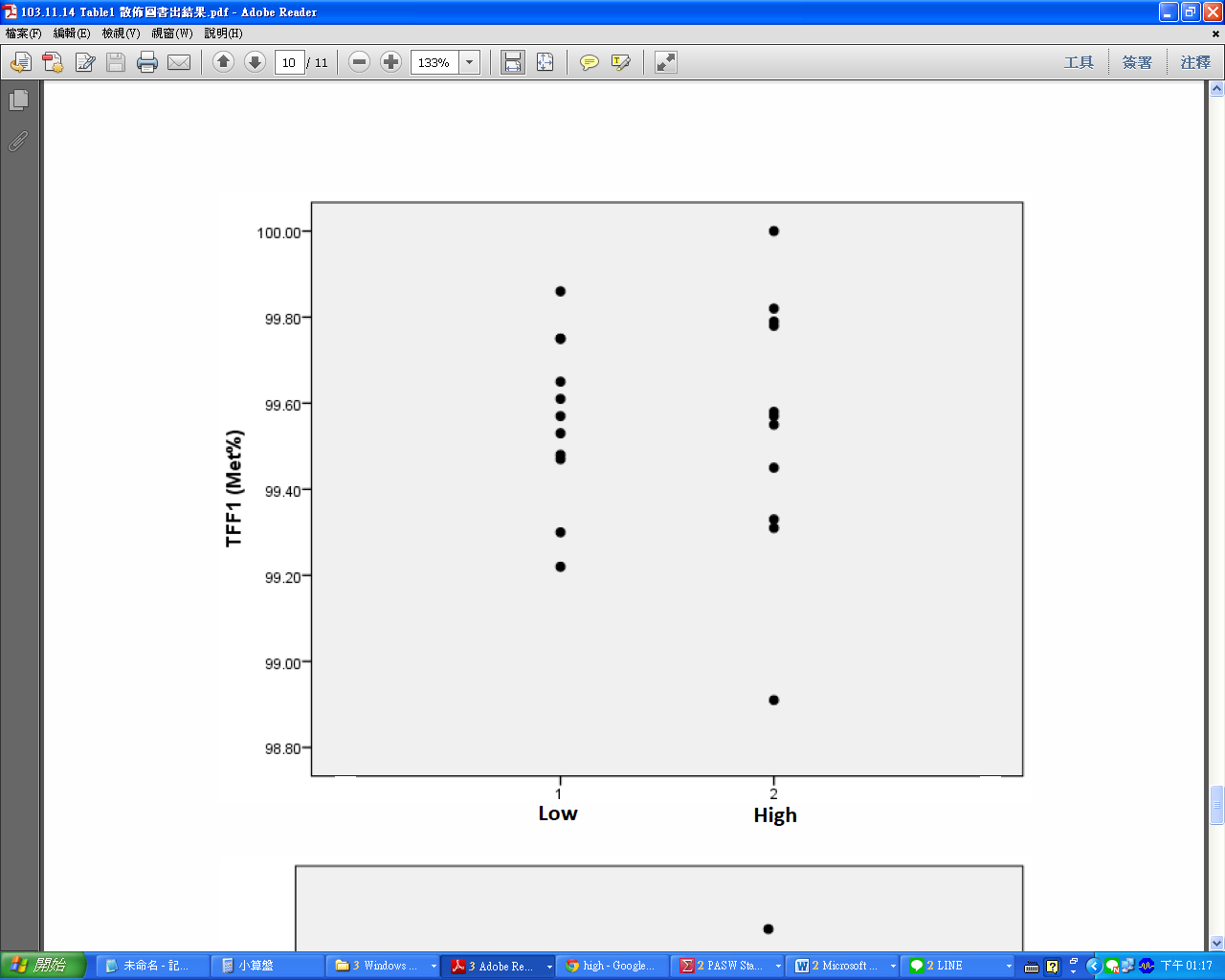 |
|  | TSS:43786644 | Low vs. high exposure  99.56±0.19 vs. 99.55±0.30  p=0.934 |  |
| **VEGFA**  **(Vascular endothelial growth factor A)**  **(7422)** | Chr6:  43737632 – 43739852 | Catalog no:335002EPHS112506-1A  CpG island location:chr6:43737632-43739852  Assay position(central point): chr6:43738844  PCR product size:168-bp  NCBI build no:37 | 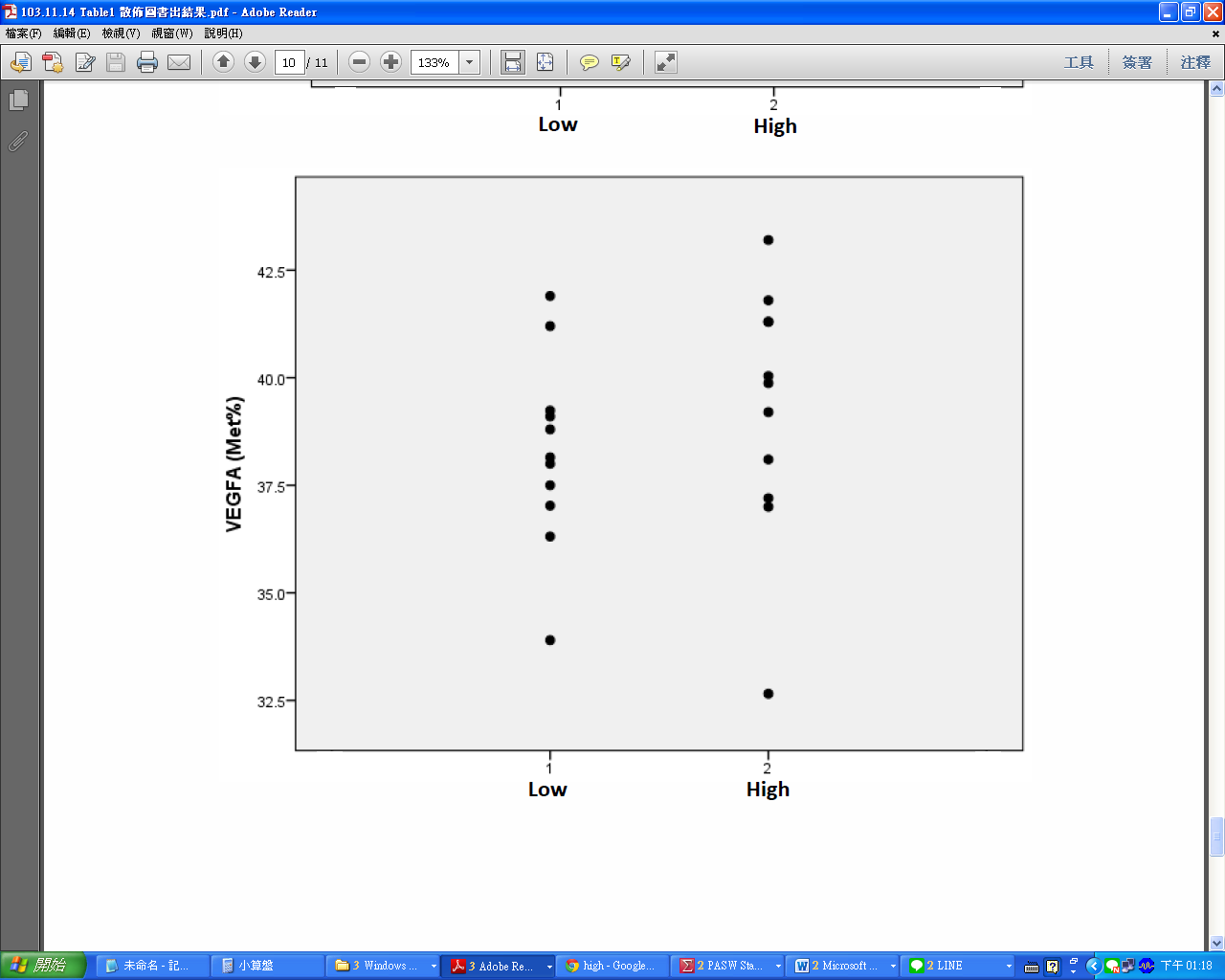 |
|  | TSS:43737945 | Low vs. high exposure  38.28±2.21 vs.39.24±2.93  p=0.396 |  |
| **MAPK1**  **(Mitogen-activated protein kinase 1)**  **(5594)** | Chr22 20443948 –  20551970 | Pyrosequence primer info:  F: GTTATTGTGTATTGTGGAGTTGA  R:Biotin- CTTTATATAACCCATTCTCCATTACTATCA  S: GTGTATTGTGGAGTTGAT  Sequence to analysis:  TYGGTGTTTTGTTTTAGTGYGGTGTTTTTTTTTGATTTTTTTATT | 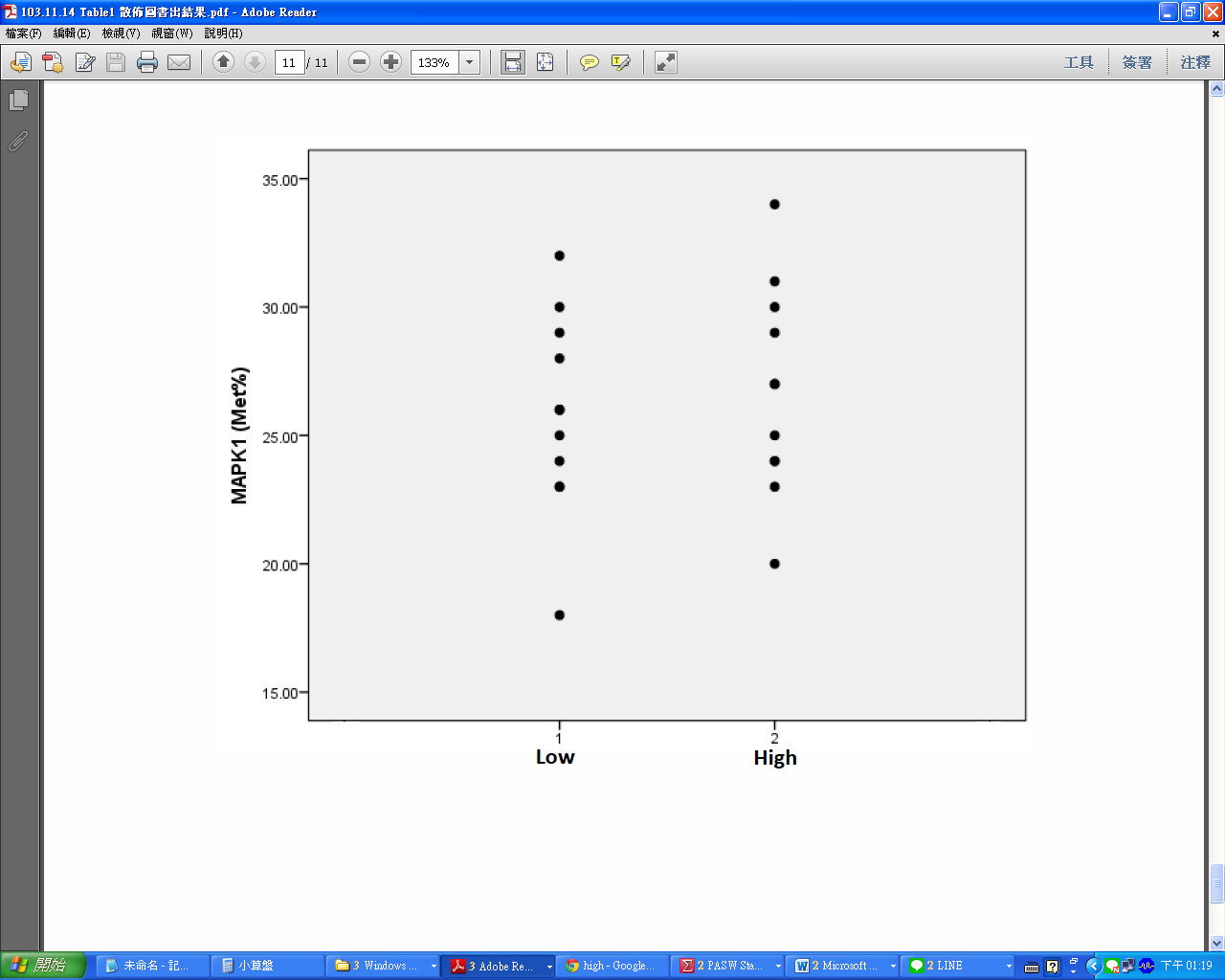 |
|  | TSS: 20441432 | Low vs. high exposure  25.82±3.89 vs. 26.73±4.05  p=0.598 |  |

**Primer Design Principles**
Each EpiTect Methyl II PCR primer assay corresponds to one distinct CpG island in a promoter region, defined as from 5 kb upstream to 3 kb downstream of transcription start site (TSS). All EpiTect Methyl II PCR primers are first designed by a rigorously optimized computer algorithm that accounts for the GC-rich sequences in genomic DNA and particularly in CpG islands. The design algorithm also insures that every amplicon contains sufficient cutting sites for both methyl-sensitive and methyl-dependent enzymes to maximize methylation detection sensitivity.

***@ TNFα* 294 base pairs PCR product sequence in** [**Table**](http://www.ncbi.nlm.nih.gov/pmc/articles/PMC2637989/table/pone-0004488-t002/) **1 is as follows:**

UCSC Genome Browser on Human Feb. 2009 (GRCh37/hg19) Assembly

>hg19_dna range=chr6:31543344-31544344 5'pad=0 3'pad=0 strand=+ repeatMasking=none

CAGACGCTCCCTCAGCAAGGACAGCAGAGGACCAGCTAAGAGGGAGAGAAGCAACTACAGACCCCCCCTGAAAACAACCCTCAGA**CG**CCACATCCCCTGACAAGCTGCCAGGCAGGTTCTCTTCCTCTCACATACTGACCCA**CG**GCTCCACCCTCTCTCCCCTGGAAAGGACACCATGAGCACTGAAAGCATGATC**CG**GGA**CG**TGGAGCTGGC**CG**AGGAGG**CG**CTCCCCAAGAAGACAGGGGGGCCCCAGGGCTCCAGG**CG**GTGCTTGTTCCTCAGCCTCTTCTCCTTCCTGAT**CG**TGGCAGG**CG**CCACCA**CG**CTCTTCTGCCTGCTGCACTTTGGAGTGAT**CG**GCCCCCAGAGGGAAGAGGTGAGTGCCTGGCCAGCCTTCATCCACTCTCCCACCCAAGGGGAAATGGAGACGCAAGAGAGGGAGAGAGATGGGATGGGTGAAAGATGTGCGCTGATAGGGAGGGATGGAGAGAAAAAAACGTGGAGAAAGACGGGGATGCAGAAAGAGATGTGGCAAGAGATGGGGAAGAGAGAGAGAGAAAGATGGAGAGACAGGATGTCTGGCACATGGAAGGTGCTCACTAAGTGTGTATGGAGTGAATGAATGAATGAATGAATGAACAAGCAGATATATAAATAAGATATGGAGACAGATGTGGGGTGTGAGAAGAGAGATGGGGGAAGAAACAAGTGATATGAATAAAGATGGTGAGACAGAAAGAGCGGGAAATATGACAGCTAAGGAGAGAGATGGGGGAGATAAGGAGAGAAGAAGATAGGGTGTCTGGCACACAGAAGACACTCAGGGAAAGAGCTGTTGAATGCCTGGAAGGTGAATACACAGATGAATGGAGAGAGAAAACCAGACACCTCAGGGCTAAGAGCGCAGGCCAGACAGGCAGCCAGCTGTTCCTCCTTTAAGGGTGACTCCCTCGATGTTAACCATTCTCCTTCTCCCCAACAGTTCCCCAGGGACCTCTCTCTAATCAGCCCTCTGG

**Figure S1.** The association between phthalate metabolites (5OH-MEHP) and TNFα DNA methylation percentage (Met%) in a larger sample of 256 children


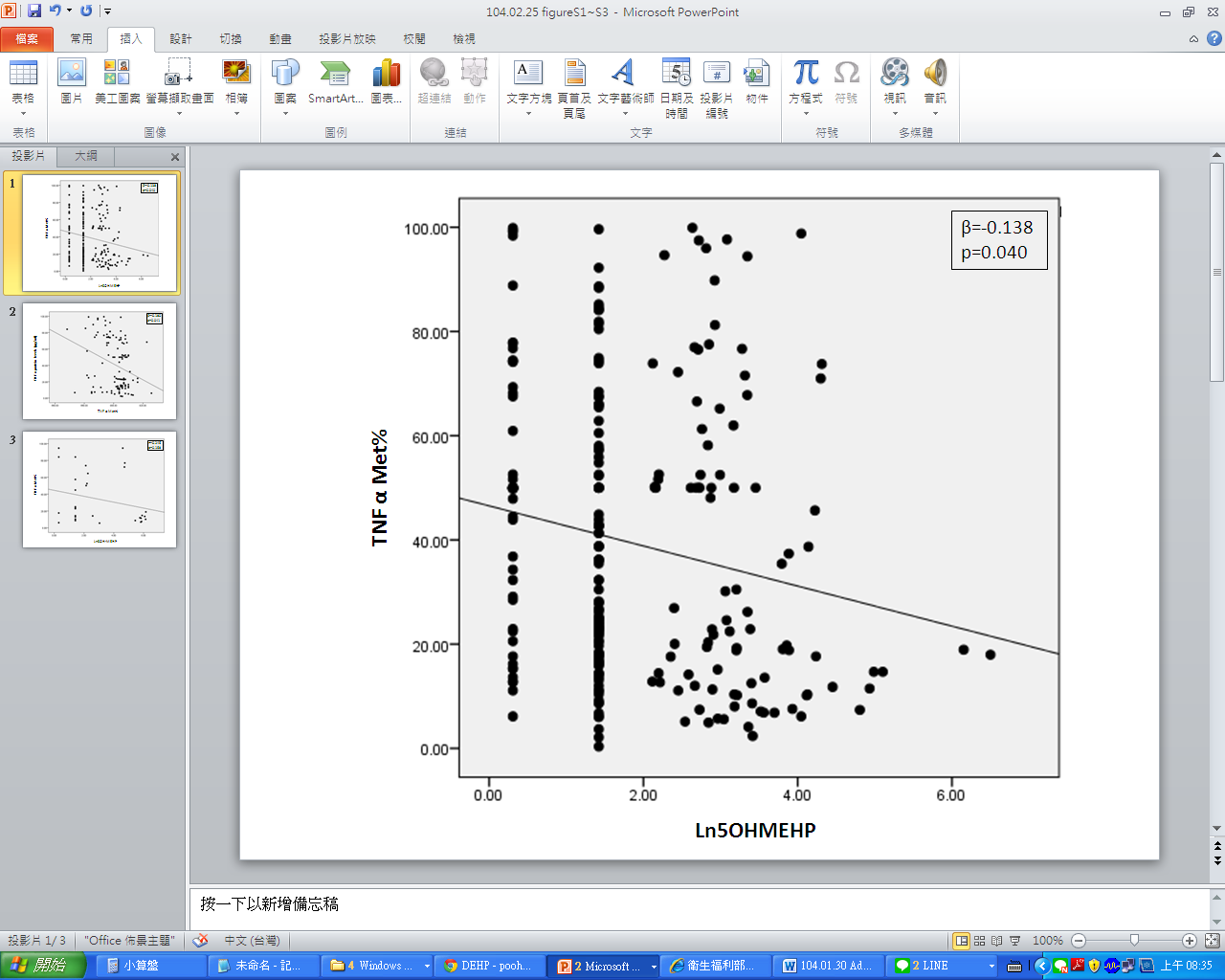


[**Figure S**](http://www.ncbi.nlm.nih.gov/pmc/articles/PMC2637989/figure/pone-0004488-g001/)**2.** The association of *TNF*α methylation percentage (Met%) and *TNF*α protein level


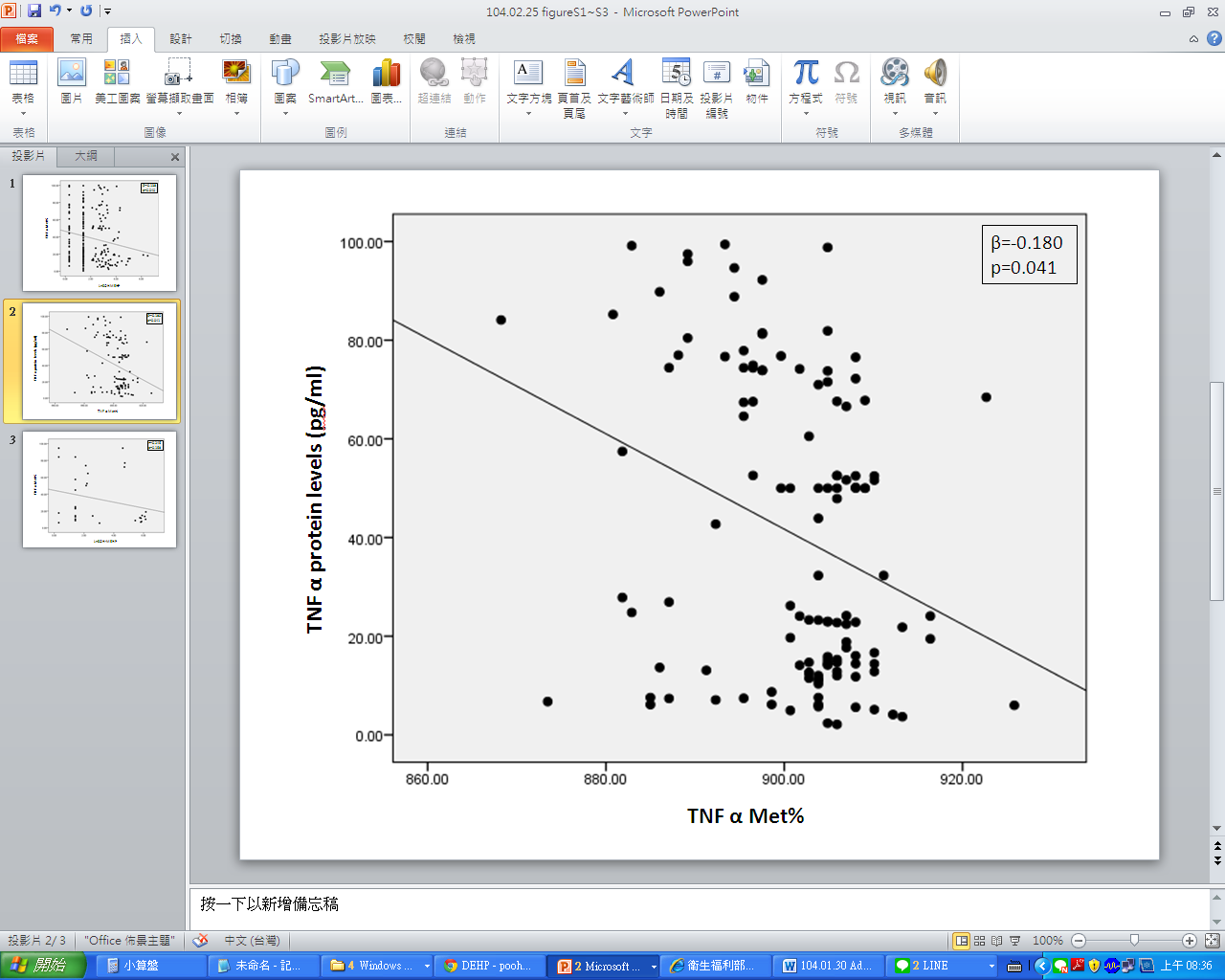


**Figure S3**. Risk ratio of asthma at age 18 years versus percent methylation of cg10717214 at

different genotypes of *TNFα* SNP rs1800610


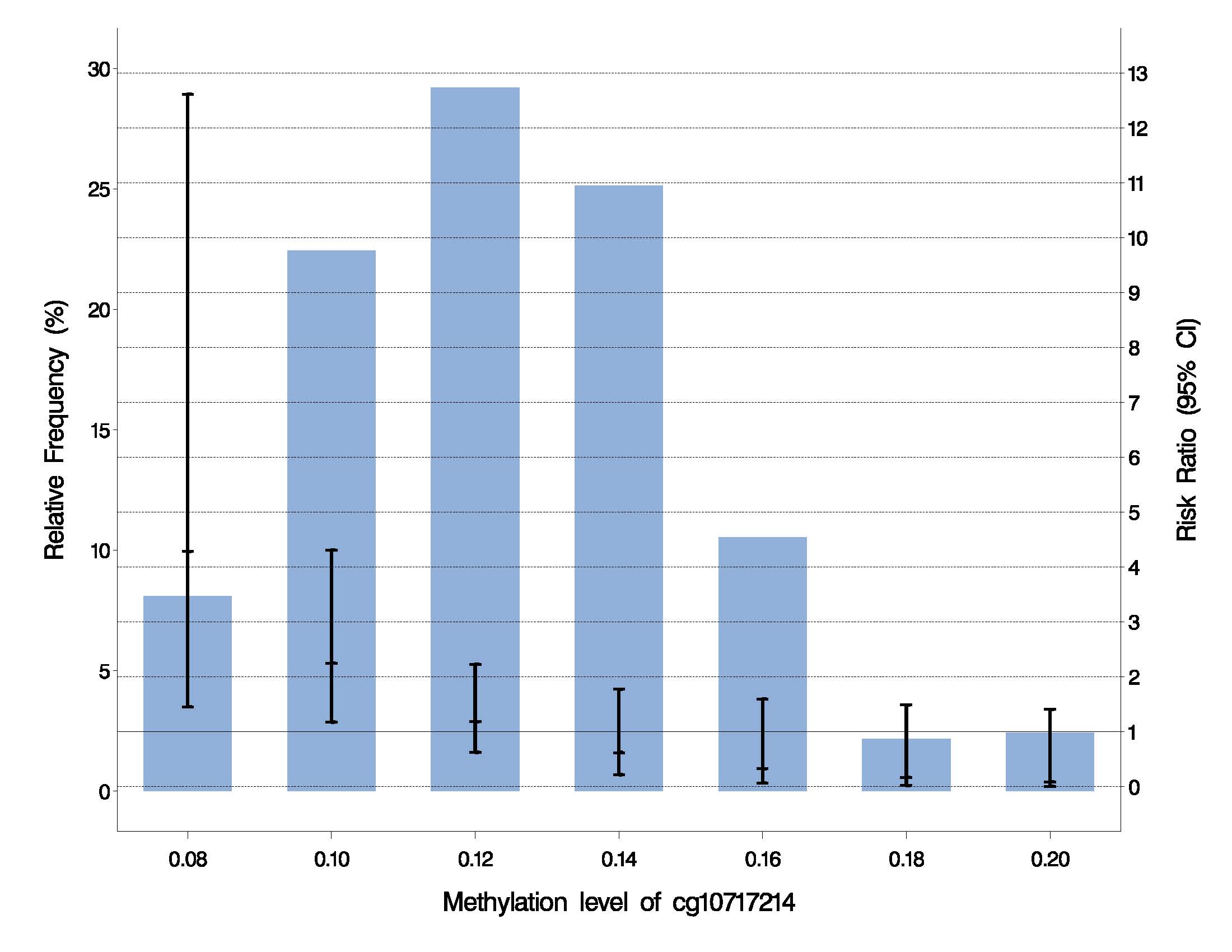


Legend

The grey-blue bars present the relative frequency of the DNA methylation levels. For instance, 8% methylation is found in 8.1% of the participants; 10% methylation is found in 22.4%. The reference genotype is “GG”. The vertical lines in the midst of the grey-blue bars show the risk ratio and the 95% confidence interval for different risk ratios with different methylation levels.

**Figure S4. Details on the mediation analysis approach**

**Figure S4(a).** **Graphical presentation of the mediation analysis.**


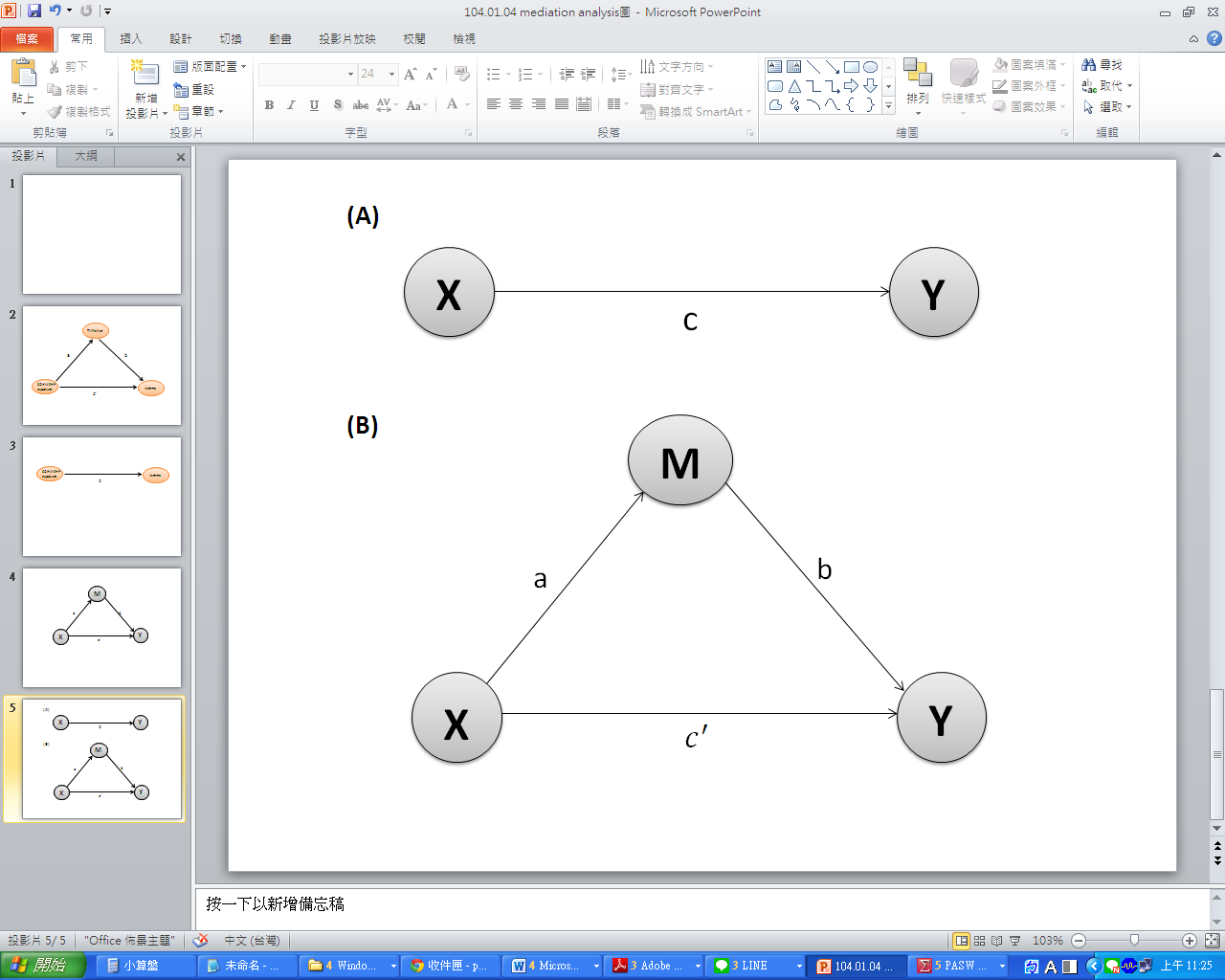


**(A) Illustration of a direct effect. Independent Variable (X) affects outcome (Y).**

**(B) Directed acyclic graphs (DAGs) depicting the possible relationships between an independent Variable (X), a potential mediator (M), and an outcome (Y).**

Mediation tests proposed by Baron and Kenny (1986) in their still immensely popular article are conducted by fitting a series of three regular (i.e., “ordinary least squares,” OLS) regressions:


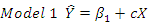


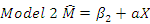


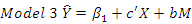


The significance of the parameter estimate c in model 1 indicates whether there exists a direct impact of X on Y (in X→Y), and model 2 and model 3 are fit to determine whether there exists an indirect effect of X on Y through the mediator M (in X→M→Y). To determine whether there is a significant mediation effect, the researcher extracts estimate a in model 2, and the estimate b in model 3.

**Figure S4(b). Steps of mediation causal analysis.**


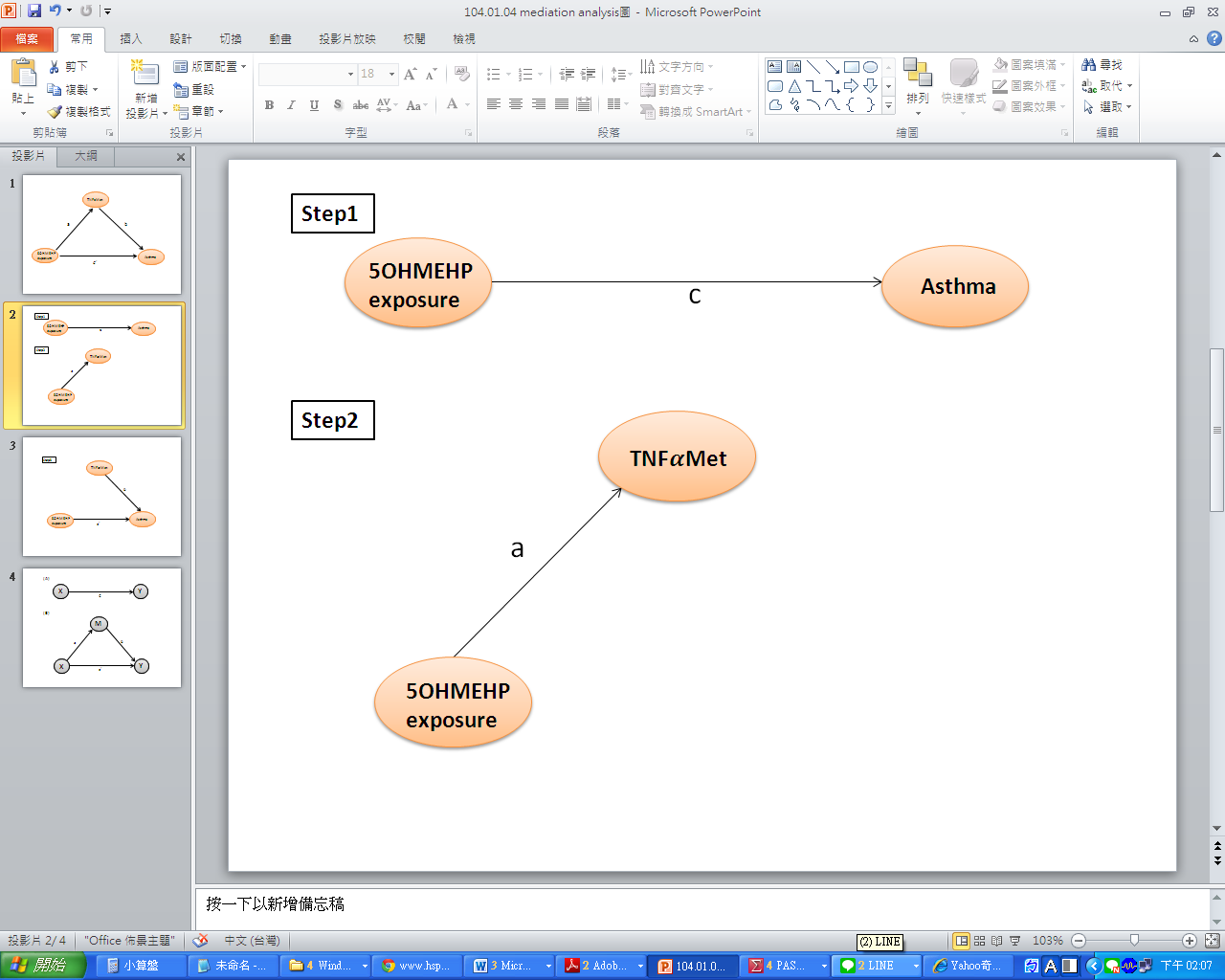


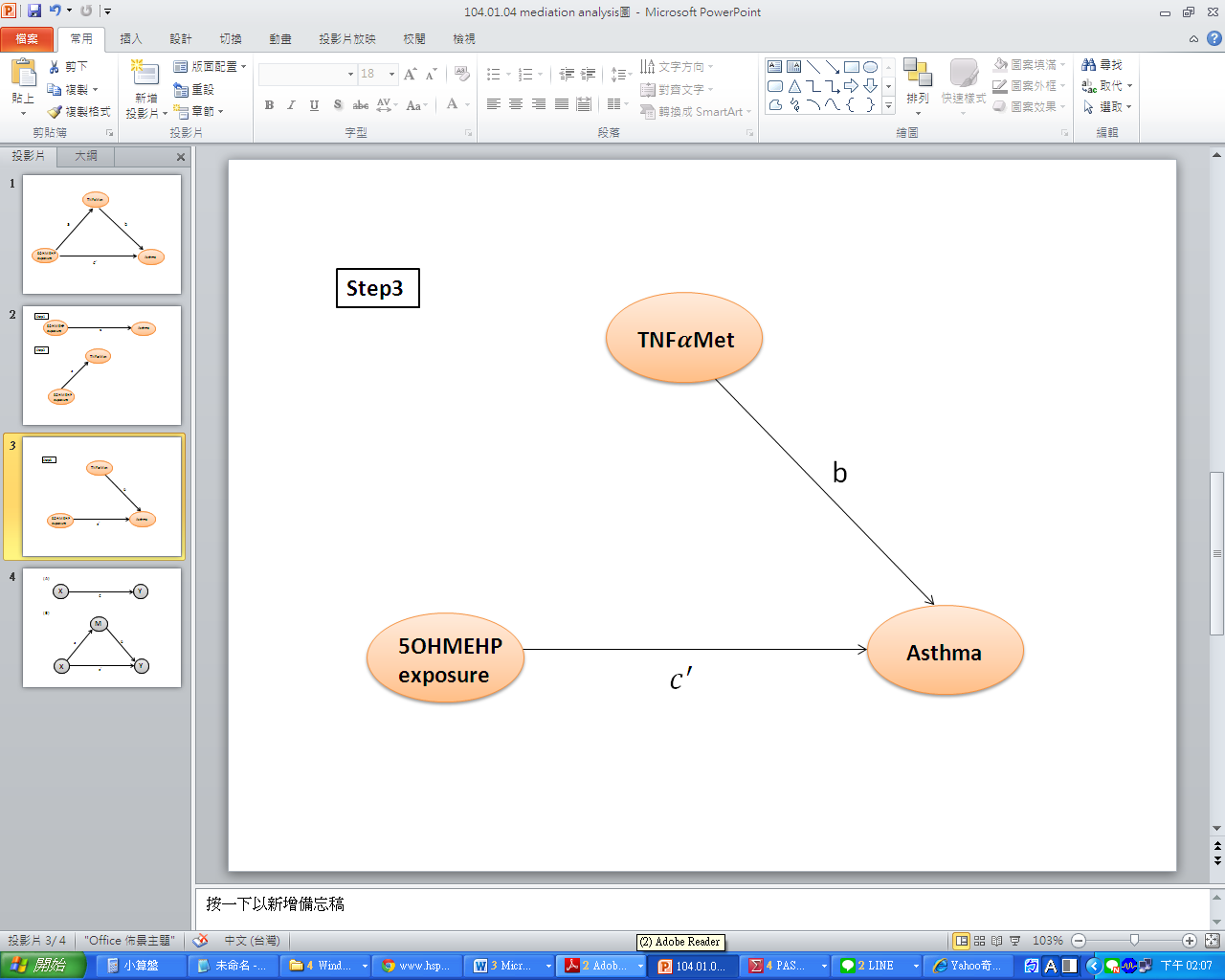


**Step 1** **Show that the independent variable (X) is correlated with the outcome (Y) (Model 1)**

**Step 2 Show that the independent variable (X) is correlated with the mediator (M) (Model 2)**

**Step 3 Show that the mediator affects the outcome variable (Model 3)**

Variables in our article are as follows:

Y: asthma (1 = No; 2=Yes)

X: 5OHMEHP exposure (1 >19.33 ng/mL; 2 <=19.33 ng/mL)

M: TNFαMet

**Step 1** **asthma→5OHMEHP exposure**


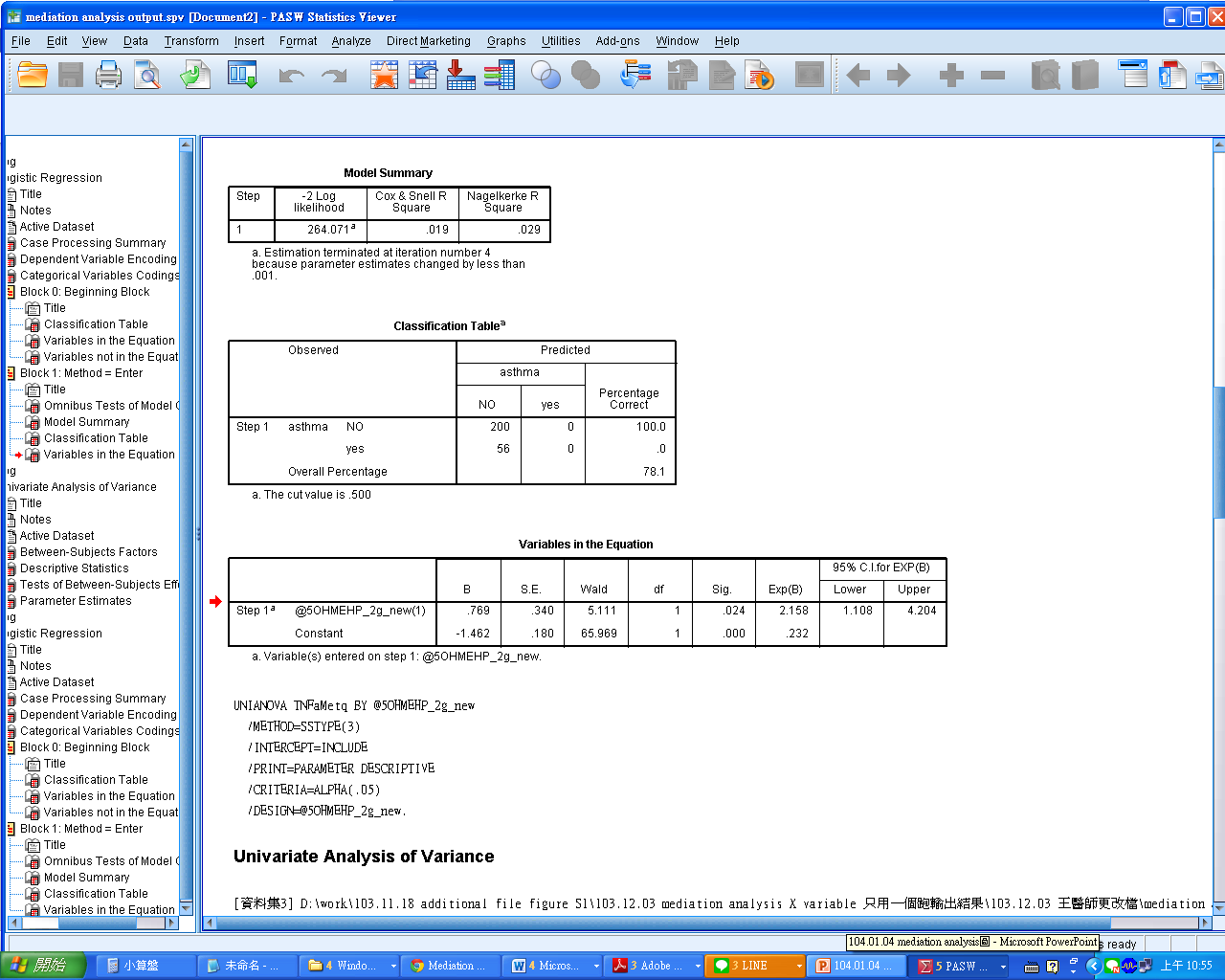


Model 1: asthma=-1.462+0.769*5OHMEHP exposure

→ c is 0.769 and p-value=0.024.

**Step 2** **TNFαMet→5OHMEHP exposure**


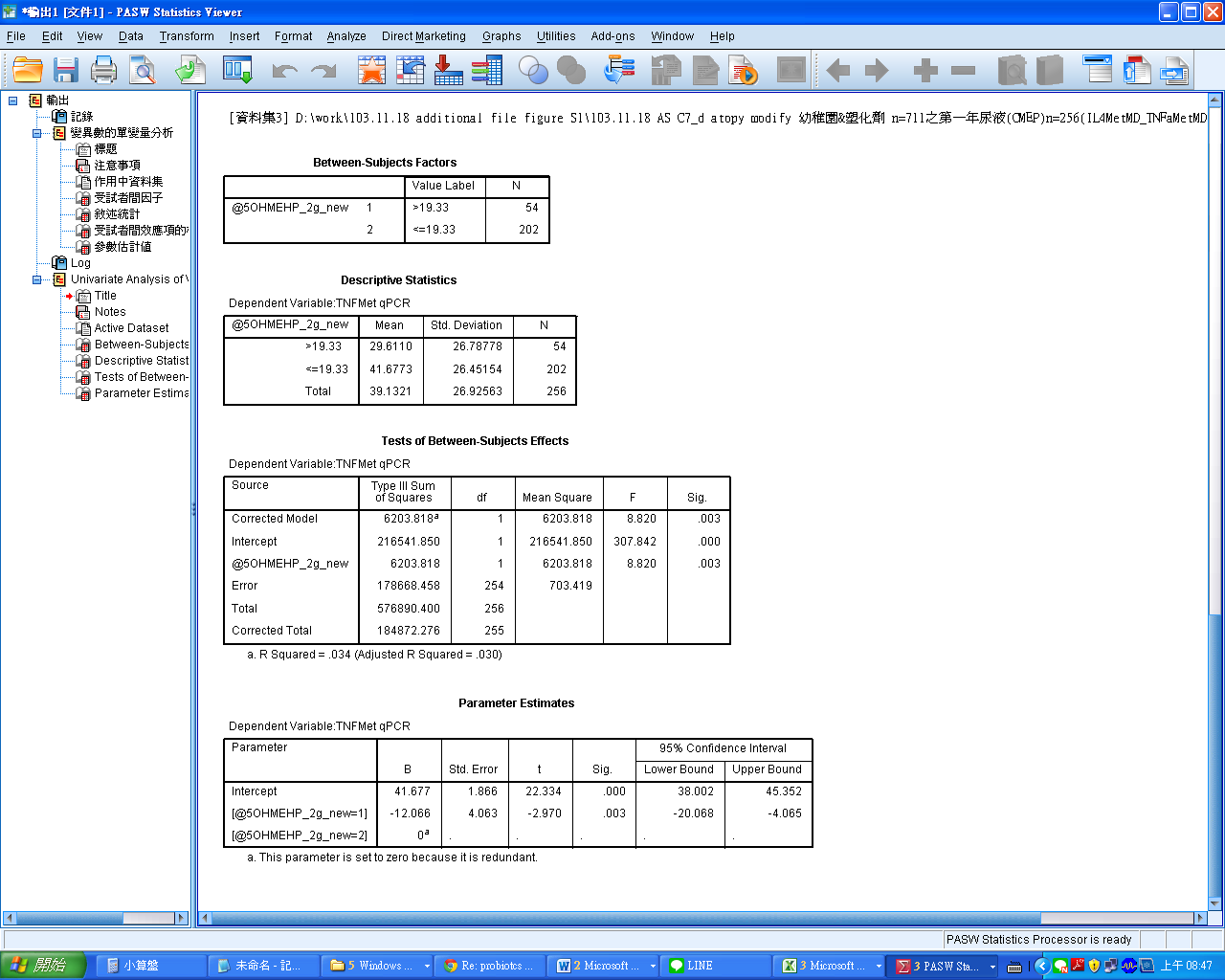


Model 2: TNFαMet=41.677-12.066*5OHMEHP exposure

→ a is -12.066 and p-value=0.003.

**Step 3** **asthma**→**5OHMEHP exposure & TNFαMet**


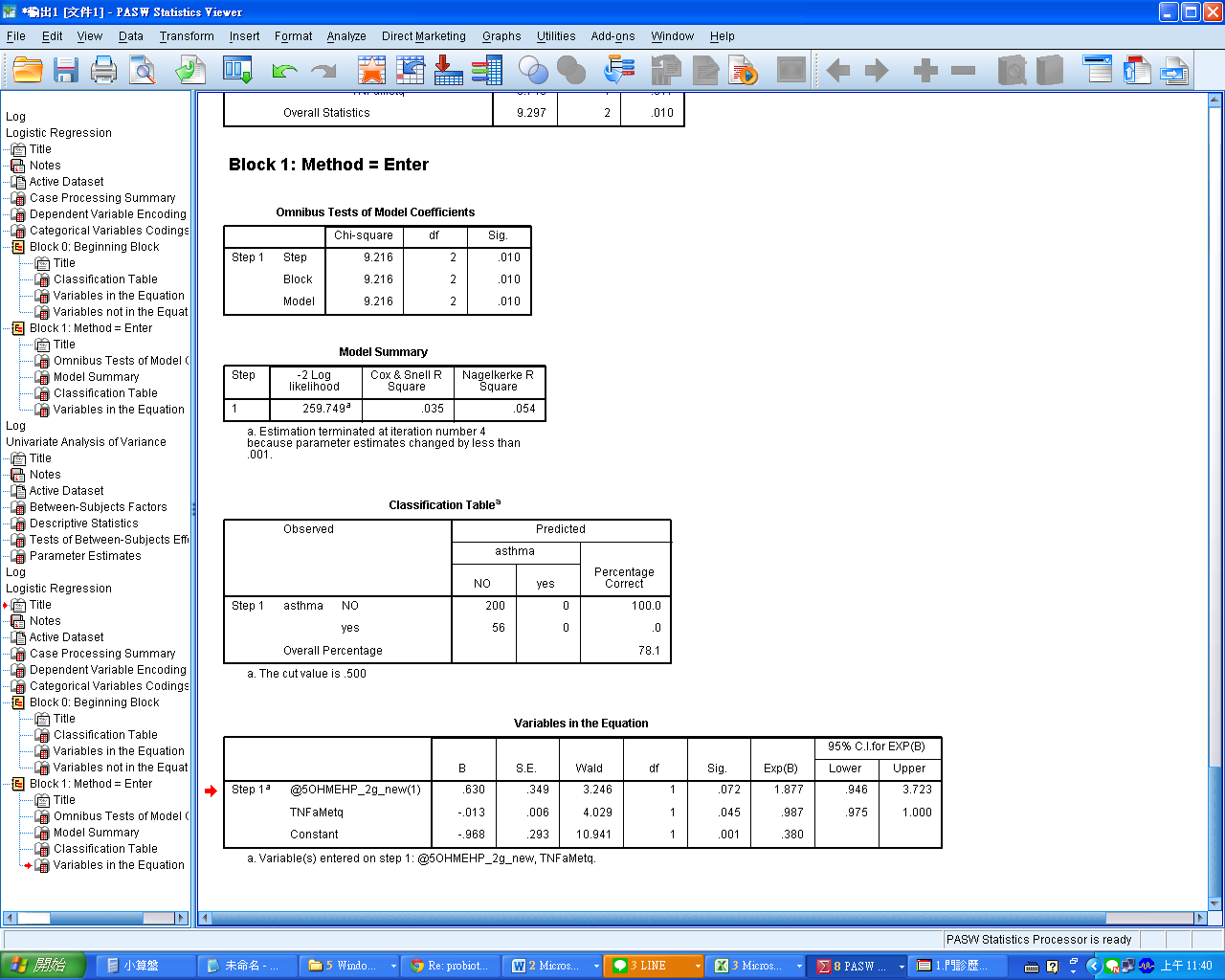


Model 3: asthma=-0.968+0.635OHMEHP exposure-0.13 TNFαMet

→b is -0.13 and p-value=0.045.

→
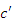
 is 0.63 and p-value=0.072.

When a, b and c is significant and
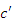
 is not significant, the medication effect is complete mediation.

**Estimation of the proportion of mediation**

The ratio of the indirect effect to the total effect


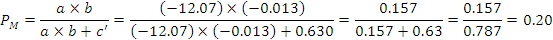


The ratio of the direct effect to the total effect


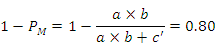


The direct mediation effect is 80%.

20% risk of asthma in relation to exposure to phthalate is mediated by changes in DNA methylation in the TNFα gene.

Hence, other pathways are likely to add to increase the mediation.

[**Figure S**](http://www.ncbi.nlm.nih.gov/pmc/articles/PMC2637989/figure/pone-0004488-g001/)**5.** The workflow chart outlining the identification of the differentially methylated

genes in a step-by-step manner by qPCR.

@ In the Pyrosequence experiment, each PyroMark Q24 plate (QIAGEN#979201) only accommodated 24 samples at one time. So that, we put 22 samples and 1 unmethylated control DNA and 1 methylated control DNA (QIAGEN#59695).

In the Real-Time PCR experiment, since each sample, included 1 unmethylated control DNA and 1 methylated control DNA (QIAGEN#59695) must division into 4 well for 4 treatments(Mo, Ms, Md, Msd). So one 96-well plate only accommodated 24-samples(included 2-control DNA). The 96well layout was below.
